# Ursolic acid ameliorates ocular surface dysfunction in dry eye via targeting EGFR/RAS/RAF/MAP2K1/MAPK1 pathway

**DOI:** 10.1016/j.jpha.2025.101294

**Published:** 2025-04-03

**Authors:** Qinghe Zhang, Ke Yan, Yufei Lv, Qiuping Liu, Yi Han, Zuguo Liu

**Affiliations:** a Department of Ophthalmology, The First Affiliated Hospital of University of South China, Hengyang Medical School, University of South China, Hengyang, Hunan, 421001, China; b Eye Institute of Xiamen University, School of Medicine, Xiamen University, Xiamen, Fujian, 361005, China

**Keywords:** Ursolic acid, Inflammation, Dry eye, Ocular surface dysfunction

## Abstract

Dry eye (DE), a multifactorial ocular surface disease, is predominantly characterized by inflammation as a central pathological factor. Ursolic acid (UA), a pentacyclic triterpenoid with well-documented anti-inflammatory properties, was evaluated in this study for its therapeutic effects on ocular surface dysfunction associated with DE and its underlying mechanisms. A hyperosmotic stress model (500 mOsM) using human corneal epithelial cells (HCEs) and an animal model of DE was established to assess UA's protective effects on both cellular and organismal levels. Comprehensive assessments, including phenol-red cotton tests and slit-lamp examinations, were performed to evaluate ocular surface damage in the DE mouse model. Potential UA-related targets and their relevance to DE pathology were identified through database mining. Protein-protein interaction (PPI) network construction and pathway enrichment analysis using the Metascape platform highlighted core targets and signaling pathways. Molecular docking simulations using AutoDock and PyMOL further elucidated the interaction modes between UA and its targets. To validate the molecular mechanisms underlying UA's therapeutic effects, integrative analyses were conducted using single-cell sequencing data from the Single Cell Portal and RNA sequencing of tissue samples. The results demonstrated that UA eye drops significantly preserved ocular surface functional units and alleviated DE symptoms, through modulation of the epidermal growth factor receptor (EGFR)/rat sarcoma (RAS)/rapidly accelerated fibrosarcoma (RAF)/mitogen-activated protein kinase (MAPK) kinase 1 (MAP2K1)/MAPK1 signaling pathway, as supported by network pharmacological analysis. Single-cell sequencing localized the distribution of key pathway proteins to the anterior ocular segment, particularly the cornea. *In vivo* experiments confirmed the therapeutic efficacy of UA eye drops via the EGFR/RAS/RAF/MAP2K1/MAPK1 pathway. Collectively, these findings underscore the potential of UA eye drops as a promising therapeutic approach for managing ocular surface disorders in DE.

## Highlight

• UA, a natural small molecule compound, is therapeutic potential for DE.
• UA maintains homeostasis in HCEs under hyperosmotic stress.
• UA promotes recovery of damaged ocular surfaces, alleviating DE *in vivo*.
• UA mitigates lacrimal and meibomian gland dysfunction associated with DE.
• UA exerts therapeutic effects on DE via modulation of the EGFR/RAS/RAF/MAP2K1/MAPK1 signaling pathway.

## Graphical abstract

**Figure:**
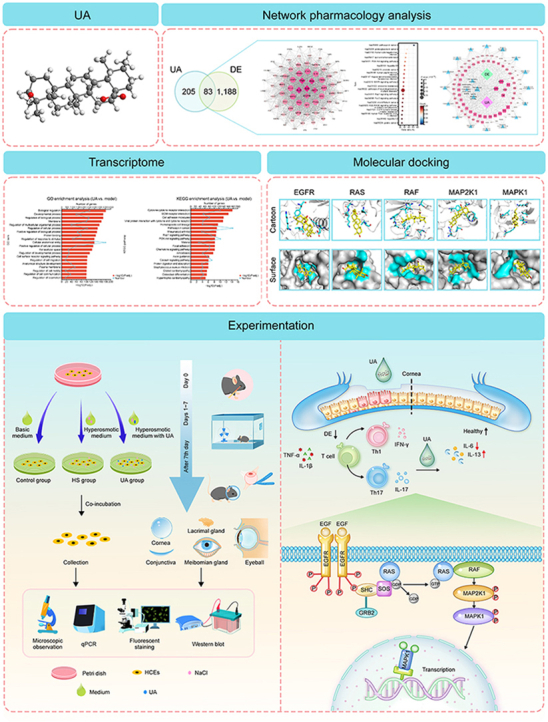

## 1 Introduction

The increasing prevalence of dry eye (DE) has been markedly influenced by the widespread use of electronic devices, deteriorating environmental conditions, and escalating life pressures [1]. DE, a multifactorial ocular surface disease, manifests through a spectrum of symptoms, including dryness and irritation [2], with inflammation serving as a critical factor in its onset and progression [3]. Current treatment options, such as artificial tears, immunosuppressants, and corticosteroids, are often limited in efficacy. Artificial tears primarily provide symptomatic relief by replenishing tear volume, while immunosuppressants are associated with notable irritation and other adverse effects [4].

Extensive phytochemical and ethnopharmacological studies have highlighted the significant anti-inflammatory potential of natural compounds derived from medicinal plants, which are associated with a reduced incidence of adverse side effects, making them promising candidates for therapeutic applications. Among these, wild chrysanthemum has demonstrated beneficial effects in the management of DE. Clinical applications of eye patches containing wild chrysanthemum (e.g., Good Vision Eye Patch, Beijing Haoshili Technology Development Co., Ltd., Beijing, China) as the primary ingredient is commonly employed to treat meibomian gland dysfunction (MGD). Ursolic acid (UA), a naturally occurring pentacyclic triterpenoid and a major bioactive component of wild chrysanthemum, exhibits a wide range of pharmacological activities, including antibacterial, antiprotozoal, anti-inflammatory, and antitumor effects [[5], [6], [7]]. In ophthalmology, UA has shown promising efficacy in mitigating the progression of diabetic retinopathy by inhibiting neovascularization. However, its specific effects on DE remain undocumented, underscoring the need for further exploration in this area.

Network pharmacology provides a computational framework for investigating biological systems, leveraging database searches to predict drug targets comprehensively [8]. By constructing a “disease-gene-target-drug” network, this approach elucidates the interconnections between targets of traditional Chinese medicine (TCM) components from a systemic perspective, offering insights into molecular interactions and potential mechanisms [9]. Potential targets of UA relevant to DE were identified using databases such as Traditional Chinese Medicine Systems Pharmacology Database and Analysis Platform (TCMSP), SwissTargetPrediction, and GeneCards. Protein-protein interaction (PPI) network analysis, along with Gene Ontology (GO) and Kyoto Encyclopedia of Genes and Genomes (KEGG) enrichment analyses conducted through Search Tool for the Retrieval of Interacting Genes (STRING) database, Metascape, and Cytoscape, identified core targets and pathways. Additionally, single-cell sequencing data were analyzed to explore the distribution of key pathway proteins within the anterior ocular segment, particularly the cornea. RNA sequencing of tissue samples further confirmed the regulatory mechanisms of the identified pathway. Both *in vivo* and *in vitro* experiments were performed to validate the therapeutic mechanism of UA. The findings revealed that UA confers therapeutic benefits for DE by stabilizing the epidermal growth factor receptor (EGFR)/rat sarcoma (RAS)/rapidly accelerated fibrosarcoma (RAF)/mitogen-activated protein kinase (MAPK) kinase 1 (MAP2K1)/MAPK1 signaling pathway, effectively repairing corneal epithelium, modulating inflammation, and mitigating DE-associated damage on the ocular surface in a murine model. UA also preserved the integrity of conjunctival goblet cells (GCs), prevented deterioration of conjunctival tissues and lacrimal glands, enhanced lipid secretion from meibomian glands, reduced abnormal lipid deposition, and facilitated the rejuvenation of both lacrimal and meibomian glands, thereby improving their overall functionality.

## 2 Materials and methods

### 2.1 Cell culture and cellular hyperosmolar stress

Human corneal epithelial cells (HCEs) were obtained from the American Type Culture Collection (ATCC) (Manassas, VA, USA) and cultured in a 5% CO_2_ incubator at 37 °C. The culture medium consisted of Dulbecco's modified Eagle medium/Nutrient Mixture F-12 (DMEM/F12) (Gibco, Carlsbad, CA, USA), supplemented with 10% fetal bovine serum (FBS) (Southern Biotech, Birmingham, AL, USA), 7.5 μg/mL insulin (Sigma-Aldrich, St. Louis, MO, USA), 10 ng/mL human epidermal growth factor (HEGF) (Sigma-Aldrich), and 1% penicillin/streptomycin (Gibco). To establish a hyperosmolar stress model, a hypertonic medium (500 mOsM) was prepared by supplementing DMEM/F12 with NaCl. HCEs were cultured in this medium for 24 h to simulate hyperosmotic stress.

### 2.2 Cell viability assay

Cell viability was evaluated using the Cell Counting Kit-8 (CCK-8) (Beyotime Biotechnology, Shanghai, China). HCEs treated with varying concentrations of UA were cultured in either normal or hypertonic medium for 12 or 24 h. Following the removal of the supernatant, 100 μL of CCK-8 working solution (100 μg/mL) was added to each well. The cells were then incubated at 37 °C in the dark for 2 h, after which optical density (OD) values were measured at 450 nm using a microplate reader.

### 2.3 Preparation of UA eye drops

UA, a pentacyclic triterpenoid carboxylic acid with the chemical name 3β-hydroxy-urs-12-*en*-28-oic acid and molecular formula C_30_H_48_O_3_, exhibits high thermal and photostability. Its hydroxyl and carboxyl functional groups contribute to diverse bioactive properties. In this study, UA powder (purity ≥ 98%; Macklin, Shanghai, China) was suspended in sterile phosphate-buffered saline (PBS) (Solvay, Alpharetta, GA, USA) and sonicated at 96 Hz and 45 °C overnight to facilitate dissolution. The resulting masterbatch was diluted to the desired concentration and stored at 4 °C.

### 2.4 Establishment of DE murine model

C57BL/6 mice (8–12 weeks old) used to establish the DE model were obtained from the Animal Centre of the University of South China (Hengyang, China). All animal experiments adhered to the Association for Research in Vision and Ophthalmology (ARVO) Statement for the Use of Animals in Ophthalmology and Vision Research and were approved by the Ethics Committee for Laboratory Animals of the University of South China (Approval No.: 2024-241). The DE model was induced through desiccation stress by housing female mice in a dry cabinet with a relative humidity of 30% ± 5%, a temperature of 20–25 °C, and continuous ventilation via an electric fan for seven days. A 12 h light-dark cycle was maintained, and the mice were fed a standard diet. To enhance desiccation stress, 0.075% benzalkonium chloride (BAC) eye drops (Sigma-Aldrich) were administered once daily, and scopolamine hydrobromide (0.25 g/100 mL; Macklin) was injected subcutaneously four times a day.

### 2.5 Topical UA administration

Experimental C57BL/6 mice were divided into groups: the control group was housed in a barrier environment without treatment, while the experimental groups were exposed to desiccation stress in a desiccator with continuous air blowing for seven days. UA eye drops were administered starting from day one, with the vehicle group receiving sterile PBS as a solvent control. The Restasis® group, serving as a positive control, was treated with cyclosporine A (CsA) (Allergan, Irvine, CA, USA). Each mouse received 2.5 μL of UA or CsA eye drops in both eyes, four times daily, for seven consecutive days. UA was administered at three concentrations: 1.2 μg/mL (low concentration UA) (LC-UA), 6 μg/mL (medium concentration UA) (MC-UA), and 30 μg/mL (high concentration UA) (HC-UA).

### 2.6 Tear secretion tests

Tear secretion was measured using phenol red cotton thread (Tianjin Jingming New Technology Development Co., Ltd., Tianjin, China). The tests were conducted one day prior to and 12 h after the final eye drop treatment. Phenol red cotton threads were gently placed in the conjunctival sac using ophthalmic forceps and removed after 15 s, as timed by a stopwatch. The length of the thread stained red by absorbed tear fluid was recorded in millimeters. Following the test, mice were briefly allowed to blink to minimize overexposure and prevent ocular surface irritation.

### 2.7 Corneal sodium fluorescein staining

Following anesthesia, the ocular surface of the mice was fully exposed, and 2.5 μL of sodium fluorescein solution (1%; Macklin) was applied to the cornea. The eyelids were manually blinked 3–5 times to distribute the dye, and the surface was rinsed with PBS to remove excess fluorescein. Residual fluorescein and PBS were gently blotted from the cornea using absorbent filter paper. The corneal surface was then imaged using a slit-lamp imaging system, and epithelial defects were assessed based on a standardized 12-point scoring system. The cornea was divided into four quadrants, each scored from 0 to 3: no staining (score 0), 1–30 punctate stains (score 1), more than 30 punctate stains without fusion (score 2), and the presence of fused, filamentous, or ulcerated stains (score 3).

### 2.8 In vivo confocal laser corneal microscopy

The ocular surface was further examined using *in vivo* confocal laser corneal microscopy. A confocal cap coated with carbomer gel (Bausch + Lomb Corp., Vaughan, Canada) was fitted over the imaging lens. After anesthesia, the eyeballs were fully exposed, stabilized, and covered with carbomer gel. High-resolution images of the corneal layers were captured for analysis.

### 2.9 Optical coherence tomography (OCT) examination

OCT was performed to evaluate both anterior and posterior segments of the eye. The anesthetized mice were positioned with their eyes fully exposed and securely fixed on a platform, ensuring stable head alignment with the OCT imaging device. Topamax phenylephrine eye drops (Bausch + Lomb Corp.) were used to dilate the pupils. Posterior segment OCT images, centered on the optic papilla, and anterior segment images, centered on the pupil, were obtained using the OPTOPROBE system (HealthOlight Technology Co., Ltd., Beijing, China).

### 2.10 Fundus imaging and fluorescence fundus angiography

Fundus imaging and fluorescence fundus angiography were performed using the OPTOPROBE system. After anesthesia, the eyes of the mice were fully exposed, and pupils were dilated with tropicamide phenylephrine eye drops. Fundus vascular images were captured, centered on the optic papilla. For fluorescence fundus angiography, mice received an intraperitoneal injection of 10% fluorescein sodium prior to imaging.

### 2.11 Histological section

Whole mice eyeballs, including the upper and lower conjunctival sacs and eyelids, were harvested, fixed in 10% paraformaldehyde and processed either for OCT embedding or paraffin embedding. OCT-embedded samples were sliced at 5-μm thickness and stored at −80 °C. For paraffin embedding, the tissue underwent fixation, dehydration, clearing, wax infiltration, and embedding, followed by sectioning into 5 μm-thick slices, which were stored at room temperature.

### 2.12 TdT-mediated dUTP nick-end labeling (TUNEL) staining

TUNEL staining (Wuhan Servicebio Technology Co., Ltd., Wuhan, China) was performed according to the manufacturer's protocol to detect apoptotic cells. Slides were counterstained with 4′,6-diamidino-2-phenylindole (DAPI) (Vector Laboratories, Burlingame, CA, USA) and visualized using a fluorescence microscopy system (Leica DM2500; Leica, Wetzlar, Germany). TUNEL-positive cells were quantified using ImageJ software.

### 2.13 Oil red O staining and LipidTOX staining

Oil red O staining (Solarbio, Beijing, China) and LipidTOX neutral lipid staining (Thermo Fisher Scientific Inc., Waltham, MA, USA) were carried out according to the respective manufacturers' protocols. After staining, lipid distribution in the lacrimal and meibomian glands was imaged using a light microscope (Nikon, Melville, NY, USA) and quantified with ImageJ software.

### 2.14 Histochemical staining

Histological analysis was conducted using hematoxylin and eosin (H&E) staining kits (Beyotime Biotechnology) and periodic acid-Schiff (PAS) staining kits (Sigma-Aldrich). Tissue sections were stained as per the manufacturers' instructions and imaged with a light microscope (Nikon). The number of PAS-stained GCs in the conjunctiva was counted and subjected to statistical analysis.

### 2.15 Immunofluorescence staining

Tissue slices were fixed with paraformaldehyde at room temperature for 10 min, followed by three washes with PBS. Sections were permeabilized using an immunofluorescence permeabilization buffer (Beyotime Biotechnology) for 10 min and washed again with PBS. Blocking was performed with an immunofluorescence blocking solution (Beyotime Biotechnology) for 60 min at room temperature. Subsequently, the sections were incubated overnight at 4 °C with primary antibodies, including anti-matrix metalloproteinase-3 (MMP-3) (1:200, Cat. No.: YT4465; Immunoway, Suzhou, China), anti-EGFR (1:200, Cat. No.: A23381; ABclonal, Wuhan, China), anti-RAS (1:200, Cat. No.: A19779; ABclonal), anti-RAF (1:200, Cat. No.: A19638; ABclonal), anti-p-MAP2K1 (1:200, Cat. No.: AP1349; ABclonal), anti-p-MAPK1 (1:200, Cat. No.: AP0974; ABclonal), anti-cleaved caspase-8 (1:200, Cat. No.: NB100-56116; Novus Biologicals, Littleton, CO, USA), anti-Ki67 (1:200, Cat. No.: A23722; ABclonal), anti-mucin 5AC (MUC5AC) (1:200, Cat. No.: YN0880; Immunoway), anti-cluster of differentiation 3 (CD3) (1:200, Cat. No.: ab135372; Abcam, Cambridge, UK), anti-tumor necrosis factor alpha (TNF-α) (1:200, Cat. No.: ab183218; Abcam), and anti-CD4 (1:200, Cat. No.: ab183685; Abcam). The next day, the sections were washed with PBS and incubated with Alexa Fluor 488-conjugated goat anti-rabbit IgG (1:300, Cat. No.: AS053; ABclonal) for 1 h at room temperature in the dark. After washing, the sections were counterstained with DAPI, stored at 4 °C in the dark, and imaged using a fluorescence microscope (Leica DM2500).

For cell-based immunofluorescence, cells were seeded onto glass coverslips in 24-well plates at a density of 1 × 10^5^ cells/well and incubated with various formulations for 24 h. Following fixation and blocking, the cells were incubated overnight at 4 °C with primary antibodies, including anti-cleaved caspase-8 (1:200, Cat. No.: NB100-56116; Novus Biologicals) and anti-Ki67 (1:200, Cat. No.: A20018; ABclonal). The next day, the coverslips were washed with PBS and incubated with Alexa Fluor 488-conjugated goat anti-rabbit IgG (1:300, Cat. No.: AS053; ABclonal) for 1 h at room temperature in the dark. After staining, the cells were counterstained with DAPI and imaged using a fluorescence microscope (Leica DM2500).

### 2.16 Quantitative reverse-transcription polymerase chain reaction (qRT-PCR)

For RT-PCR, tissue samples were lysed using TRIzol® reagent (Takara Bio, Shiga, Japan) and homogenized with a tissue grinder (JXFSTPRP-CL; Shanghai Jingxin Industrial Development Co., Ltd, Shanghai, China). Total RNA was extracted and reverse transcribed into complementary DNA (cDNA) using the HiScript III All-in-One RT SuperMix Perfect for qPCR (Vazyme, Nanjing, China). qPCR was performed using the StepOne Plus Real-Time PCR detection system (Applied Biosystems, Darmstadt, Germany) and ChamQ Universal SYBR qPCR Master Mix (Vazyme). β-actin served as the internal control. The relative expression levels of target genes were calculated using the comparative cycle threshold (*C*_*T*_) method (fold change = 2^–ΔΔ^^*C*^*T*). Primer sequences used in the analysis are listed in Table 1.

**Table 1:** Primers for quantitative reverse transcription polymerase chain reaction (qPCR).

Gene	Forward sequence (5′–3′)	Reverse sequence (5′–3′)
*h-β-actin*	ACAGAGCCTCGCCTTTGC	GCGGCGATATCATCATCC
*h-IL-6*	CCAGAGCTGTGCAGATGAGT	ATTTGTGGTTGGGTCAGGGG
*h-IL-17*	CCCCTAGACTCAGGCTTCCT	AGTTCGTTCTGCCCCATCAG
*h-IL-13*	TGACCACGGTCATTGCTCTC	GATTCCAGGGCTGCACAGTA
*m-β-actin*	CCTAAGGCCAACCGTGAAAAG	AGGCATACAGGGACAGCACAG
*m-MMP-3*	CCTTTTGATGGGCCTGGAAC	GAGTGGCCAAGTTCATGAGC
*m-IL-6*	CCCCAATTTCCAATGCTCTCC	CGCACTAGGTTTGCCGAGTA
*m-IL-17*	CGCAATGAAGACCCTGATAGAT	CTCTTGCTGGATGAGAACAGAA
*m-IFN-γ*	AAATCCTGCAGAGCCAGATTAT	GCTGTTGCTGAAGAAGGTAGTA
*m-NOS2*	GCCCAGCCAGCCCAAC	GCAGCTTGTCCAGGGATTCT

*h*-*β-actin*: human β-actin; *h*-*IL-6*: human interleukin-6; *m*-*β-actin*: mouse-β-actin; *m*-*MMP-3*: mouse matrix metalloproteinase-3; *m*-*IL-6*: mouse IL-6; *m*-*IFN-γ*: mouse interferon gamma; *m*-*NOS2*: mouse nitric oxide synthase 2.

### 2.17 Western blot

Drug-treated HCEs were collected, and the mice cornea and conjunctiva were carefully dissected, washed, and placed in tubes containing radioimmunoprecipitation assay (RIPA) buffer (Thermo Fisher Scientific Inc.) for protein lysis. Extracted proteins were quantified and loaded into gel lanes for electrophoresis before being transferred to a polyvinylidene fluoride (PVDF) membrane. The membrane was washed and incubated overnight with primary antibodies, including vinculin (1:10000, Cat. No.: 13901S; Cell Signaling Technology, Danvers, MA, USA), EGFR (1:2000, Cat. No.: A11351; ABclonal), RAS (1:2000, Cat. No.: A19779; ABclonal), RAF (1:2000, Cat. No.: A19638; ABclonal), p-MAP2K1 (1:2000, Cat. No.: 2338; Cell Signaling Technology), MAP2K1 (1:2000, Cat. No.: A4868; ABclonal), p-MAPK1 (1:2000, Cat. No.: AP0974; ABclonal), MAPK1 (1:2000, Cat. No.: A4782; ABclonal), interleukin-6 (IL-6) (1:2000, Cat. No.: ab290750; Abcam), TNF-α (1:1000, Cat. No.: ab183218; Abcam), IL-1β (1:2000, Cat. No.: ab216995; Abcam), nuclear factor kappaB (NF-κB) (1:2000, Cat. No.: A22331; ABclonal), and p-NF-κB (1:1000, Cat. No.: 3033; Cell Signaling Technology). Following incubation, the membrane was washed three times and treated with horseradish peroxidase (HRP)-conjugated goat anti-rabbit IgG (1:5000, Cat. No.: a0545; Sigma-Aldrich) or HRP-conjugated mouse anti-goat IgG (1:5000, Cat. No.: a9452; Sigma-Aldrich) for 1 h. Protein bands were visualized using the Ultra Chemiluminescence Reagents Kit (Shanghai Epizyme Biomedical Technology Co., Ltd., Shanghai, China) and captured using the ChemiDoc XRS System (BioRad, Hercules, CA, USA). Densitometric analysis of the bands was conducted using ImageJ software.

### 2.18 Target prediction for DE and UA

UA targets were predicted using PharmMapper [10], TCMSP [11], SwissTargetPrediction [12], and HERB [13]. DE targets were identified using DrugBank [14], GeneCards [15], Online Mendelian Inheritance in Man (OMIM) [16], GeneMAP [17], and Therapeutic Target Database (TTD) [18]. The identified targets were normalized and converted to gene names using the UniProt database to ensure consistency. Duplicate values within UA and DE target sets were removed, and their intersections were identified using a Venn diagram. The overlapping targets were subsequently visualized using Cytoscape v.3.9.0 software.

### 2.19 Analyse PPI networks

PPI data were retrieved from the STRING database [19], with a minimum confidence score threshold set at 0.04. The PPI network was constructed using Cytoscape v.3.9.0 to identify key central targets.

### 2.20 GO and KEGG enrichment analyses

GO and KEGG pathway enrichment analyses were conducted on the intersecting targets using the Metascape [20] and Database for Annotation, Visualization and Integrated Discovery (DAVID) [21] platforms. GO analysis included molecular function (MF), cellular component (CC), and biological process (BP) categories. The top 20 enriched terms were visualized for further interpretation.

### 2.21 Drug-disease-target-pathway network construction and molecular docking

The drug-disease-target-pathway network was constructed by integrating core targets and pathways associated with UA and DE into Cytoscape v.3.9.0 for visualization and analysis.

### 2.22 Molecular docking

Molecular docking simulations were performed using AutoDock Vina 1.1.2 to evaluate the binding affinity of UA (PubChem CID: 64945) with proteins EGFR (UniProt ID: Q01279), MAPK1 (UniProt ID: P63085), MAP2K1 (UniProt ID: P31938), RAF (UniProt ID: Q99N57), and RAS (UniProt ID: P32883). Protein pre-processing, including the removal of water molecules and redundant ligands and the addition of hydrogen atoms, was conducted using PyMOL v.2.4. PDBQT files for docking simulations were generated with AutoDock Tools v.1.5.6. The docking results provided the top nine binding poses, with the conformation exhibiting the lowest binding energy and highest clustering frequency considered to be the most probable interaction. Docking results were visualized using PyMOL v.2.4.

### 2.23 Single cell sequencing database analysis and RNA sequencing

Single-cell sequencing data was obtained from the Single Cell Portal database [22] and analyzed using online tools. Total RNA was extracted from corneas using TRIzol® reagent, followed by RNA purification, reverse transcription, library construction, and sequencing, performed by Majorbio Bio-pharm Biotechnology Co., Ltd. (Shanghai, China) according to standard protocols. Functional analysis results were visualized using online platforms.

### 2.24 Statistical analysis

Statistical analyses were conducted using GraphPad Prism software (GraphPad Software, San Diego, CA, USA). A significance threshold of *P* < 0.05 was applied for all tests. Parametric comparisons of means across multiple groups were performed using one-way analysis of variance (ANOVA) or Welch ANOVA with the Dunnett test, and data were presented as mean ± standard error of the mean (SEM). Non-parametric comparisons were conducted using the Kruskal-Wallis test for medians, with results expressed as median ± interquartile range (IQR). All *P*-values were two-sided.

## 3 Results

### 3.1 UA maintains HCEs homeostasis under hyperosmotic stress

**Fig. 1:**
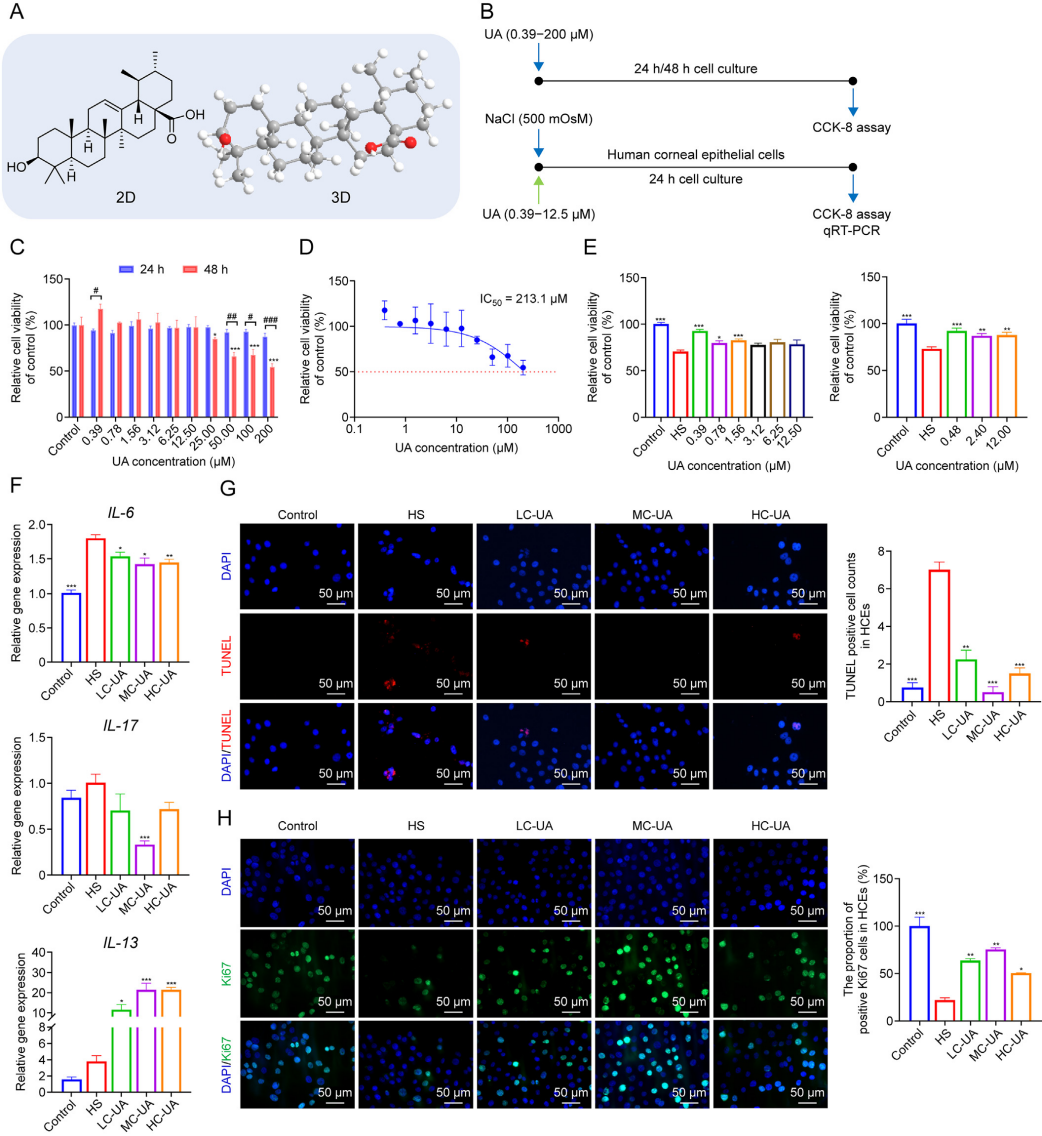
Ursolic acid (UA) effectively restores human corneal epithelial cells (HCEs) homeostasis under hypertonic stress (HS). (A) Two-dimensional (2D) and 3D chemical structures of UA. (B) Schematic representation of the *in vitro* study design. (C) Relative viability of HCEs treated with UA for 24 or 48 h. (D) Half maximal inhibitory concentration (IC_50_) value of UA on HCEs after 48 h. (E) Relative cell viability of HCEs treated with varying UA concentrations under hyperosmotic conditions (500 mOsM) for 24 h. (F) Expression levels of interleukin-6 (*IL-6*), *IL-17*, and *IL-13* in HCEs under hyperosmotic conditions after treatment with different UA concentrations for 24 h. (G) Representative images (left) and fluorescence intensity quantification (right) of TdT-mediated dUTP nick-end labeling (TUNEL) staining in HCEs treated with UA under hyperosmotic conditions for 24 h (*n* = 4 per group). (H) Representative images (left) and fluorescence intensity quantification (right) of Ki67 staining in HCEs treated with UA under hyperosmotic conditions for 24 h (*n* = 3 per group). Data are presented as mean ± standard error of the mean (SEM). ^∗^*P* < 0.05, ^∗∗^*P* < 0.01, and ^∗∗∗^*P* < 0.001 for comparisons with the model group; ^#^*P* < 0.05, ^##^*P* < 0.01, and ^###^*P* < 0.001 for comparisons of relative cell viability between 24 and 48 h treatments. CCK-8: Cell Counting Kit-8; qRT-PCR: quantitative reverse transcription polymerase chain reaction; HS: hypertonic stress; LC-UA: low concentration of UA; MC-UA: medium concentration of UA; HC-UA: high concentration of UA; DAPI: 4′,6-diamidino-2-phenylindole.

The chemical structural formula, three-dimensional (3D) representation of UA, and the study design of cell safety and efficacy assessments are illustrated (Figs. 1A and B). To evaluate the effects of UA on HCEs, various UA concentrations were applied during cell culture. After 24 and 48 h of incubation, a CCK-8 assay was performed to determine the time-dependent impact of UA concentration on HCEs viability. The results indicated no significant differences in HCEs viability after 24 h of incubation with UA concentrations ranging from 0.39 to 200 μM. However, after 48 h of incubation, while cell viability remained unaffected at concentrations between 0.39 and 200 μM, a significant reduction was observed within the range of 25–200 μM. The half maximal inhibitory concentration (IC_50_) of UA was calculated at 213.1 μM, indicating that although 200 μM UA inhibited HCEs viability to some extent, UA maintained a favorable safety profile (Figs. 1C and D). To simulate the conditions of DE, a hyperosmotic model of HCEs was established using a culture medium with an osmotic pressure of 500 mOsM. This model enabled the evaluation of UA's effects at varying concentrations and exposure durations on HCEs viability via the CCK-8 assay. Under hyperosmotic conditions, a marked reduction in cell viability was observed. Notably, UA concentrations ranging from 0.39 to 12.5 μM significantly preserved cell viability, suggesting a protective effect of UA against hyperosmotic-induced cellular damage (Fig. 1E). For further analysis, three UA concentration groups, LC-UA (0.48 μM), MC-UA (2.4 μM), and HC-UA (12 μM), were carefully selected from this range for subsequent experiments. IL-6 and IL-17, key inflammatory mediators involved in the pathogenesis of DE, were evaluated. PCR analysis demonstrated significant upregulation of these inflammatory factors following the culture of HCEs in the hyperosmotic medium. The introduction of UA significantly mitigated the production of pro-inflammatory cytokines, including IL-6 and IL-17, while promoting the secretion of the anti-inflammatory cytokine IL-13, highlighting the robust anti-inflammatory potential of UA (Fig. 1F). By employing hyperosmotic stress to mimic the pathological conditions of DE, apoptosis in HCEs was successfully induced. Apoptosis is characterized by chromosomal DNA fragmentation, including double-strand and single-strand breaks, which are absent in non-apoptotic cells. Consequently, TUNEL staining was utilized to detect apoptotic cells (Fig. 1G). Further investigations assessed cleaved caspase-8, a pivotal cysteine-aspartate protease implicated in apoptosis (Fig. S1), and Ki67, a marker of cellular proliferation, through immunostaining [[23], [24], [25]]. The results demonstrated that UA effectively attenuated hyperosmotic stress-induced apoptosis, evidenced by reduced cleaved caspase-8 staining, while concurrently promoted cellular proliferation, as indicated by increased Ki67 expression (Fig. 1H).

### 3.2 UA reliefs DE *in vivo*

Before evaluating the therapeutic efficacy of UA eye drops in an animal model of DE, a thorough safety assessment was performed. Mice received topical administration of UA eye drops at the intended concentrations for seven consecutive days (Fig. S2). Slit-lamp photography revealed that the corneas of mice treated with UA eye drops retained transparency comparable to the control group, with no signs of fluorescent staining detected (Fig. S3). Further validation using advanced imaging modalities, including anterior segment OCT and *in vivo* confocal laser corneal microscopy, confirmed that UA eye drops caused no detectable damage to the ocular surface (Figs. S4 and S5). To ensure a comprehensive safety profile, intraocular structures, particularly the retina, were examined. Fundus photography and retinal OCT analyses revealed no adverse effects on retinal integrity (Fig. S6). Additionally, histopathological evaluation via H&E staining substantiated the absence of tissue abnormalities, underscoring the excellent safety profile of UA eye drops and their suitability for ocular use (Fig. S7).

**Fig. 2:**
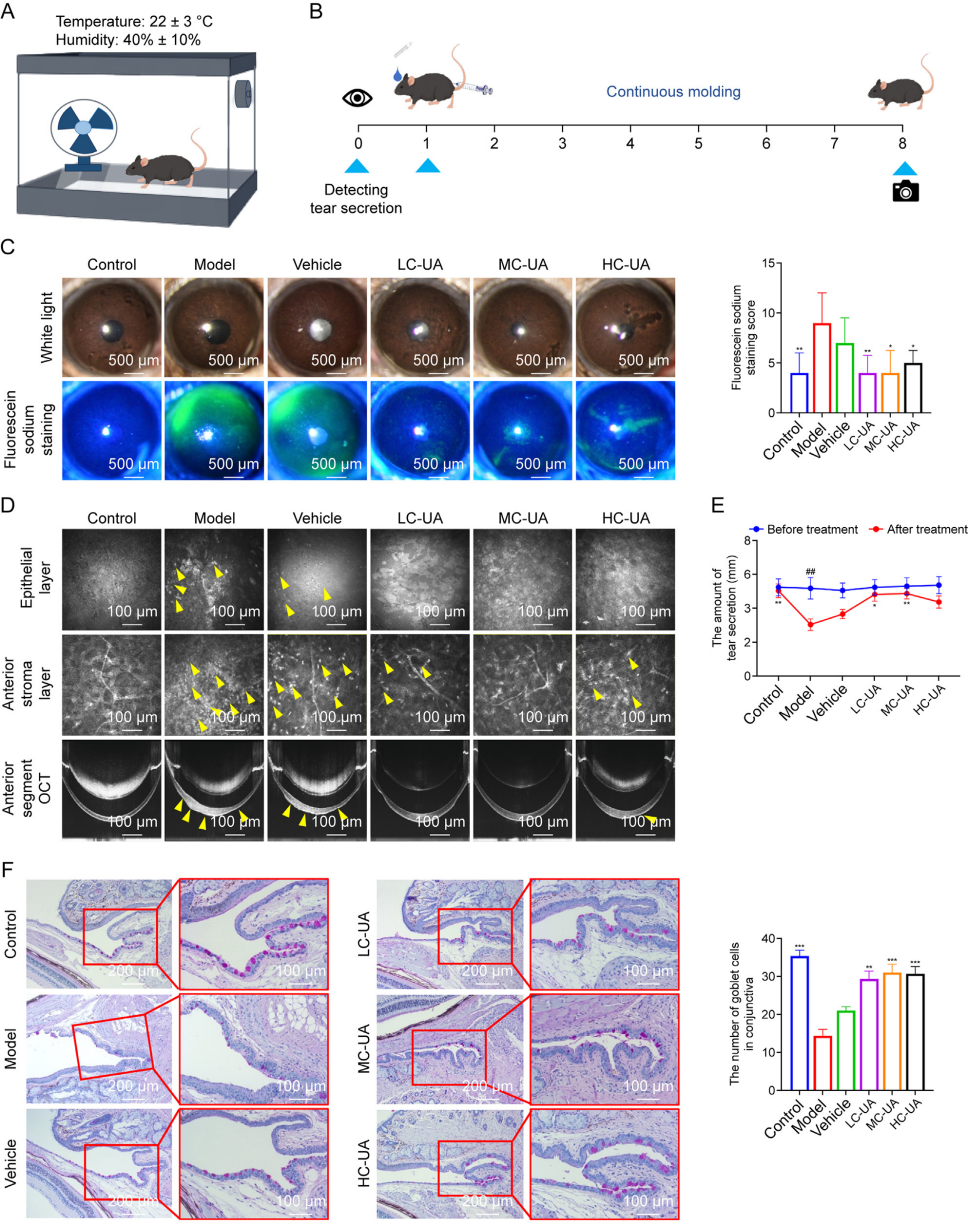
Ursolic acid (UA) effectively alleviates dry eye (DE) symptoms *in vivo*. (A) Schematic representation of the mouse model establishment. (B) Timeline of the experimental procedures for mouse model development. (C) Corneal fluorescein sodium staining images (left) and quantification scores (right) under slit-lamp examination (*n* = 12 per group). (D) Representative *in vivo* confocal laser corneal microscopy and anterior segment optical coherence tomography (OCT) images. (E) Tear secretion measurements (*n* = 8–12 per group). (F) Periodic acid-Schiff (PAS) staining of conjunctival goblet cells (GCs) (left) with corresponding quantification (right) (*n* = 6 per group). Data in Fig. 2C are presented as median ± interquartile range (IQR), while other data are expressed as mean ± standard error of the mean (SEM). ^∗^*P* < 0.05, ^∗∗^*P* < 0.01, and ^∗∗∗^*P* < 0.001, comparison between the indicated group and the model group following benzalkonium chloride (BAC) and UA eye drop application; ^##^*P* < 0.01, comparison within the same group before and after BAC and UA eye drop application. LC-UA: low concentration of UA; MC-UA: medium concentration of UA; HC-UA: high concentration of UA.

DE is a multifaceted condition, with mixed DE being particularly prevalent. This subtype arises from a combination of mechanisms involving both aqueous deficiency and evaporative factors, which, while interrelated, do not fully overlap. To rigorously assess the therapeutic efficacy of UA eye drops in managing DE, a mouse model mimicking ocular surface damage characteristic of DE was established. This was achieved through topical application of 0.075% BAC and subcutaneous scopolamine injections in a controlled, dry, and ventilated environment (Figs. 2A and B). Corneal epithelial staining and tear production were utilized as key metrics to evaluate the severity of DE. The model group displayed pronounced corneal epithelial fluorescent staining, indicative of severe epithelial damage, compared to the control group. However, topical application of UA eye drops significantly reduced fluorescent staining across all treatment groups. Notably, LC-UA and MC-UA treatments exhibited the most pronounced efficacy, comparable to CsA eye drops, though residual epithelial defects persisted at higher UA concentrations, potentially attributable to drug toxicity (Figs. 2C and S8A). Further insights into corneal integrity were obtained through OCT, which revealed irregularities and significant variations in corneal epithelial thickness in the model group compared to the control group. Treatment with UA or CsA eye drops resulted in a smoother corneal surface and more uniform thickness. *In vivo* confocal laser corneal microscopy corroborated these results, showing sparse, irregularly sized, and reduced numbers of corneal epithelial cells in the model group, which significantly improved following UA eye drop treatment. Additionally, the model group exhibited activated corneal anterior stromal cells with enhanced cytosolic reflections and substantial inflammatory cell infiltration, which were markedly reduced by UA eye drop application, indicating significant attenuation of inflammation (Figs. 2D and S8B). Tear secretion measurements aligned with these observations, showing a significant reduction in tear production in the model group compared to the control group. This reduction was effectively reversed by UA eye drop treatment (Fig. 2E), with no statistically significant differences in efficacy observed between UA and CsA eye drop therapies (Fig. S8C). Furthermore, conjunctival GCs, essential for mucin secretion and tear film stability, were markedly depleted in both the model and vehicle groups. Treatment with UA eye drops significantly ameliorated this depletion, as demonstrated by PAS staining (Fig. 2F). The improvement in GC restoration and anti-inflammatory effects achieved by UA was comparable to that of CsA, underscoring the potent therapeutic potential of UA in managing DE (Fig. S8D).

### 3.3 UA enhances the recovery process of corneal injury caused by DE

**Fig. 3:**
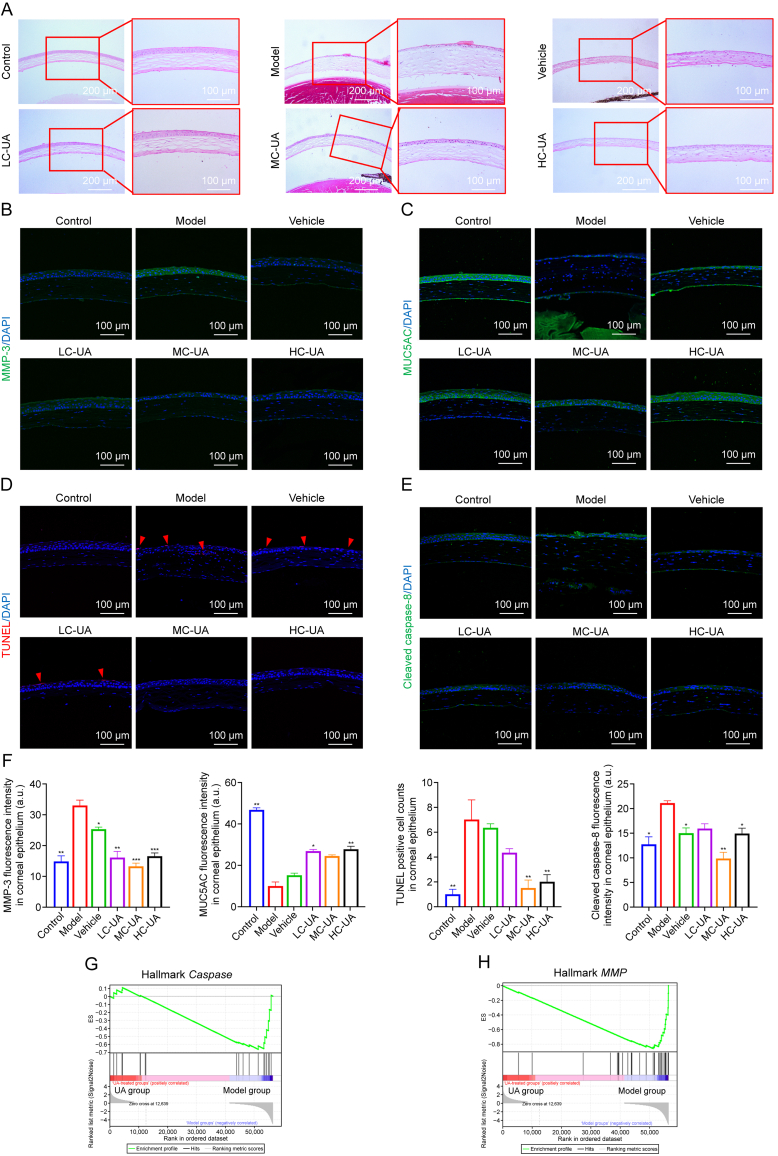
Ursolic acid (UA) protects the cornea from desiccating stress. (A) Corneal hematoxylin and eosin (H&E) staining. (B) Immunofluorescence staining of matrix metalloproteinase-3 (MMP-3) in the cornea. (C) Immunofluorescence staining of mucin 5AC (MUC5AC) in the cornea. (D) Corneal TdT-mediated dUTP nick-end labeling (TUNEL) staining. (E) Immunofluorescence staining of cleaved caspase-8 in the cornea. (F) Quantification of fluorescence intensity for MMP-3, MUC5AC, TUNEL-positive cell counts, and cleaved caspase-8 (*n* = 3–5 per group). (G) Transcriptomic gene set enrichment analysis (GSEA) analysis of *Caspases*. (H) Transcriptomic GSEA analysis of *MMPs*. Data are presented as mean ± standard error of the mean (SEM). ^∗^*P* < 0.05, ^∗∗^*P* < 0.01, and ^∗∗∗^*P* < 0.001, comparison between the specified group and the model group. LC-UA: low concentration of UA; MC-UA: medium concentration of UA; HC-UA: high concentration of UA; DAPI: 4′,6-diamidino-2-phenylindole; ES: enrichment score.

The cornea serves as the primary defensive barrier for the eye, with its structural integrity playing a critical role in preventing pathogen infiltration. The organized and uniform arrangement of corneal stromal fibers is essential for maintaining visual clarity, while densely packed corneal cells, free from inflammatory infiltration, characterize normal corneal morphology. In the model group, the corneal epithelium displayed a disrupted structure characterized by loosened organization and heightened inflammatory cell infiltration, consistent with findings reported in previous studies. The onset of DE induces significant imbalance, leading to damage and detachment of the superficial epithelial layer, as well as loosening of the corneal stroma. These structural changes confirm the successful establishment of a DE model. Additionally, the model exhibited increased vacuole formation and substantial infiltration of inflammatory cells. In contrast, treatment with UA eye drops markedly improved the ocular surface, restoring the epithelium to an intact and smooth state without observable defects or inflammatory cell infiltration (Fig. 3A). The barrier function of the corneal epithelium, evaluated through qPCR analysis of gene MMP-3, revealed that DE compromised tight junction integrity, reducing the barrier's efficacy (Fig. S9). Immunofluorescence staining corroborated these results, demonstrating a reduction in MMP-3 expression after UA treatment (Fig. 3B). These results underscore UA's protective role in preserving corneal epithelial barrier integrity by suppressing MMP-3 expression. Tears, composed of aqueous, mucin, and lipid layers, are vital for ocular health. MUC5AC, a critical mucin marker, is closely associated with DE pathogenesis [26]. In DE, reduced mucin levels shorten tear film breakup time, exacerbating inflammation and inducing corneal epithelial cell apoptosis, as evidenced by upregulation of TUNEL staining and cleaved caspase-8. UA treatment significantly upregulated MUC5AC expression, mitigating inflammation and apoptosis in corneal epithelial cells (Figs. 3C–F). Transcriptomic analysis further confirmed this effect, showing reduced *caspase* expression following UA eye drop application (Fig. 3G), further suggesting that UA protects the corneal epithelial barrier (Fig. 3H). Overall, these results demonstrate that UA effectively inhibits corneal apoptosis and preserves epithelial barrier function, thereby maintaining ocular surface homeostasis in DE.

### 3.4 UA reduces conjunctival inflammation and damage in DE

**Fig. 4:**
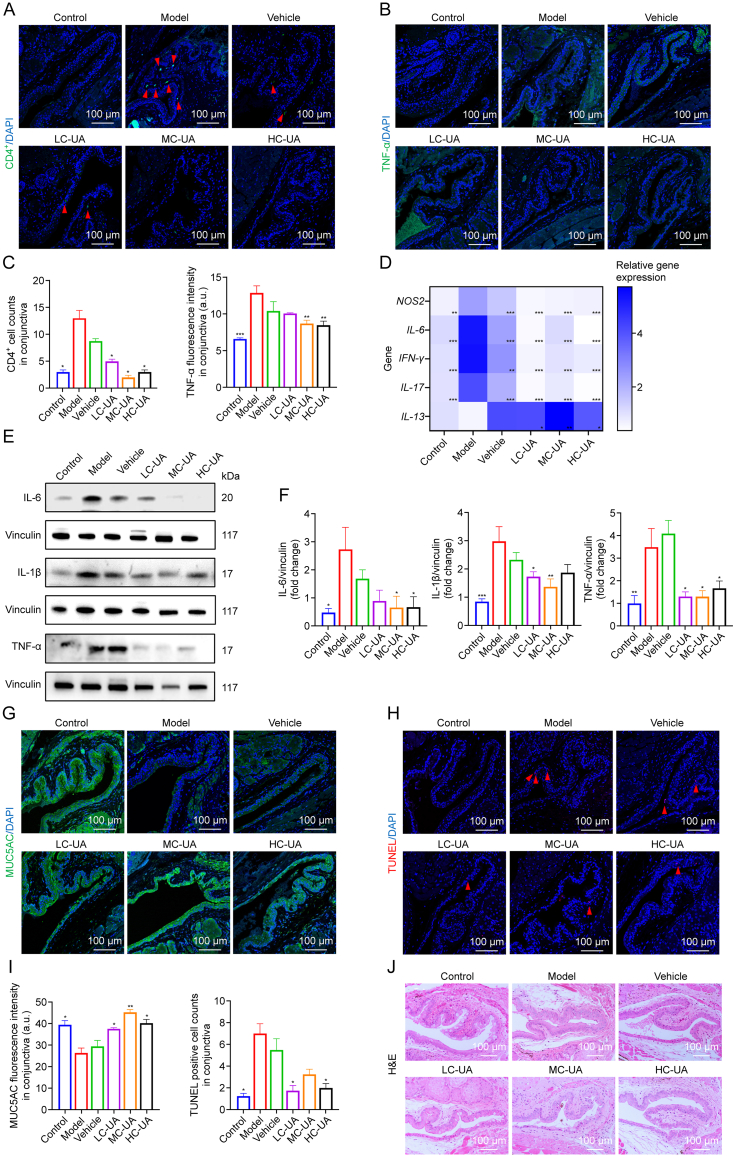
Ursolic acid (UA) alleviates conjunctival inflammation and damage associated with dry eye (DE). (A) Immunofluorescence staining of cluster of differentiation 4 (CD4^+^) cells in the conjunctiva. (B) Immunofluorescence staining of tumor necrosis factor alpha (TNF-α) in the conjunctiva. (C) Counts of CD4^+^ cells and quantification of immunofluorescence intensity for TNF-α in the conjunctiva (*n* = 4 per group). (D) Quantitative analysis of nitric oxide synthase 2 (*NOS2*), interleukin-6 (*IL-6*), interferon gamma (*IFN-γ*), *IL-17*, and *IL-13* gene expression levels in conjunctival tissues. (E) Western blot analysis of IL-6, IL-1β, and TNF-α protein levels in the conjunctiva. (F) Quantification of IL-6, IL-1β, and TNF-α protein levels in conjunctival tissues (*n* = 5–6 per group). (G) Immunofluorescence staining of mucin 5AC (MUC5AC) in the conjunctiva. (H) TdT-mediated dUTP nick-end labeling (TUNEL) staining in conjunctival tissues. (I) Quantification of immunofluorescence intensity for MUC5AC and counts of CD4^+^ cells in the conjunctiva (*n* = 3–5 per group). (J) Hematoxylin and eosin (H&E) staining of conjunctival tissues. Data are presented as mean ± standard error of the mean (SEM). ^∗^*P* < 0.05, ^∗∗^*P* < 0.01, and ^∗∗∗^*P* < 0.001, comparison between the specified group and the model group. DAPI: 4′,6-diamidino-2-phenylindole; LC-UA: low concentration of UA; MC-UA: medium concentration of UA; HC-UA: high concentration of UA.

A chronic immune response is a key driver in the pathogenesis of DE, characterized by increased infiltration of immune cells, notably CD4^+^ cells and TNF-α, into conjunctival tissue. Both the model and vehicle groups displayed significant infiltration of CD4^+^ cells and elevated TNF-α levels in conjunctival tissues. However, UA treatment markedly reduced CD4^+^ cell infiltration to levels comparable with the control group. Among the treatment concentrations, MC-UA demonstrated superior efficacy in reducing TNF-α infiltration (Figs. 4A–C). The ability of UA to suppress inflammatory factor infiltration in the conjunctiva was validated through qPCR analysis of genes such as nitric oxide synthase 2 (*NOS2*), interferon gamma (*IFN-γ*), *IL-6*, *IL-17*, and *IL-13* (Fig. 4D), as well as Western Blot detection of IL-6, IL-1β, and TNF-α protein levels (Figs. 4E and F). These inflammatory mediators are pivotal in the pathogenesis of DE. The inflammatory environment in DE is marked by the upregulation of NOS2, IFN-γ, IL-6, IL-17, IL-1β, and TNF-α, coupled with the downregulation of the anti-inflammatory cytokine IL-13. Inflammatory mediators exacerbate the condition by inducing apoptosis in conjunctival epithelial and GCs, resulting in diminished tear and mucin secretion. The subsequent destabilization of the tear film perpetuates the vicious cycle of DE. In alignment with these observations, the model group exhibited significantly decreased MUC5AC expression, reflecting mucin depletion. In contrast, UA treatment stimulated MUC5AC expression, counteracting this deficiency. TUNEL staining further demonstrated that UA eye drops effectively mitigated apoptosis in conjunctival epithelial cells (Figs. 4G–I). H&E staining highlighted conjunctival epithelial damage and GC loss in the model and vehicle groups, which were significantly ameliorated by UA treatment (Fig. 4J). These results underscore the therapeutic potential of UA in interrupting the inflammatory cascade, reducing epithelial cell apoptosis, and restoring ocular surface homeostasis in DE.

### 3.5 UA ameliorates lacrimal and meibomian glands disorder induced by DE

A comprehensive evaluation was conducted to assess the functionality and architecture of the meibomian and lacrimal glands, which play a critical role in the production of lacrimal fluid. The fluid consists of three distinct layers, aqueous, lipid, and mucin, with the aqueous layer primarily produced by the lacrimal glands and the lipid layer mainly secreted by the meibomian glands. The structural and functional integrity of these glands is crucial in the pathogenesis of DE syndrome [26]. To investigate the impact of UA eye drops on the meibomian and lacrimal glands in a murine model of DE, these glands were obtained from mice following modeling. The glands were carefully flattened between slides for microscopic analysis.

**Fig. 5:**
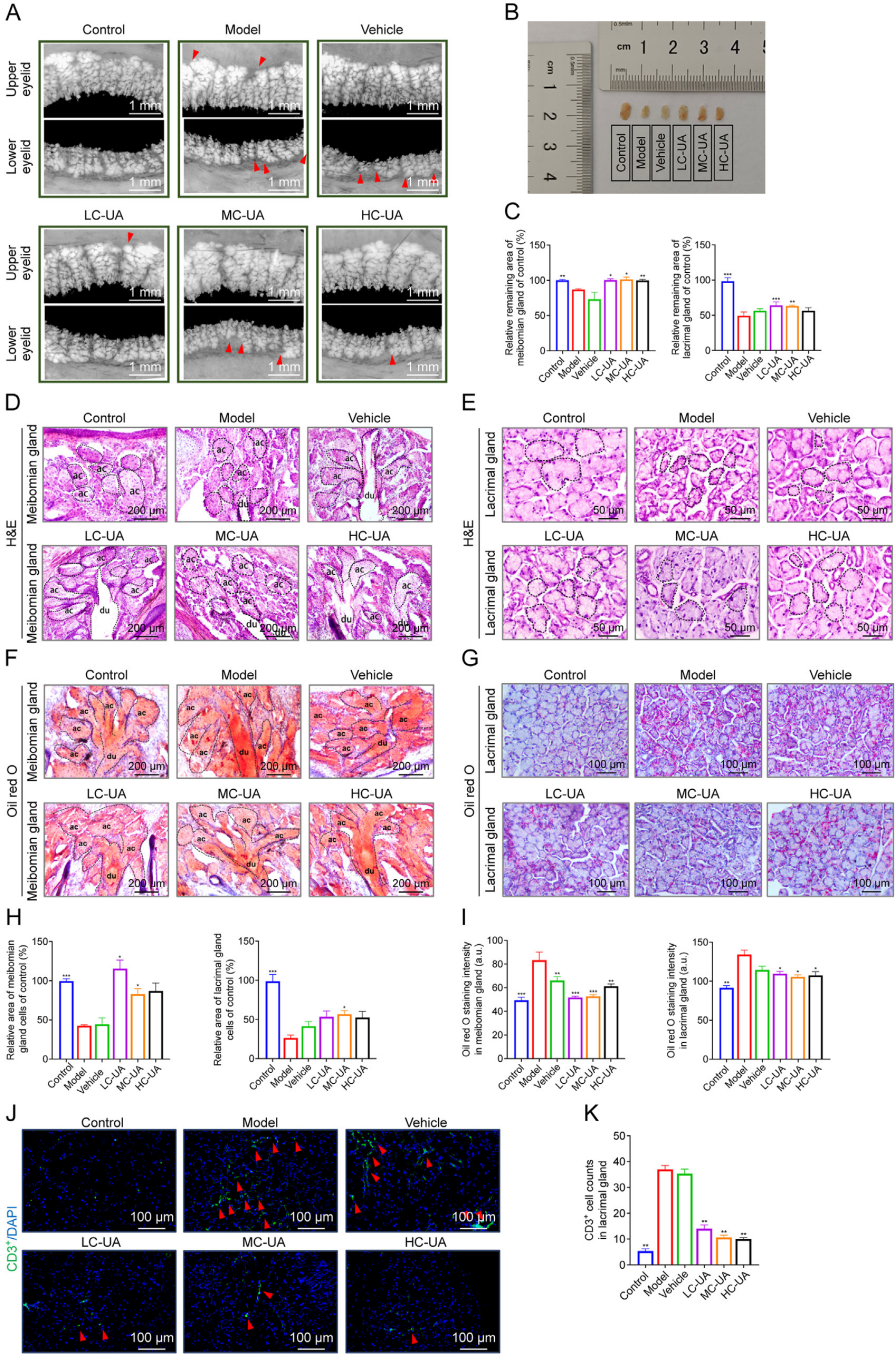
Ursolic acid (UA) ameliorates pathological conditions in the lacrimal and meibomian glands. (A) Macroscopic images of the meibomian glands under different treatment groups. (B) Macroscopic images of the lacrimal glands size under different treatment groups. (C) Quantification of the relative remaining area of meibomian and lacrimal glands (*n* = 3–4 per group). (D, E) Hematoxylin and eosin (H&E) stainings of the meibomian glands (D) and the lacrimal glands (E). (F, G) Oil red O stainings of the meibomian glands (F) and the lacrimal glands (G). (H) Quantification of the relative meibomian gland cells area and lacrimal gland cells area of control (*n* = 4–6 per group). (I) Quantification of oil red O stainings of the meibomian glands and the lacrimal glands (*n* = 4–6 per group). (J) Immunofluorescence staining of cluster of differentiation 3 (CD3^+^) cells in the lacrimal glands. (K) Quantification of CD3^+^ cells in lacrimal glands (*n* = 3 per group). Data are presented as mean ± standard error of the mean (SEM). ^∗^*P* < 0.05, ^∗∗^*P* < 0.01, and ^∗∗∗^*P* < 0.001, comparison between the indicated group and the model group. LC-UA: low concentration of UA; MC-UA: medium concentration of UA; HC-UA: high concentration of UA; ac: acinar cell; du: ductal cell; DAPI: 4′,6-diamidino-2-phenylindole.

The results demonstrated that UA eye drops significantly improved the pathological conditions of both the meibomian and lacrimal glands in mice with DE. In particular, the meibomian glands of the model group displayed pronounced deficiencies compared to the control group, which were partially restored after the administration of UA eye drops. Moreover, UA exhibited a notable capacity to mitigate lacrimal gland atrophy in dry-eyed mice. While significant atrophy was evident in the model group compared to controls, no such difference was observed in the three UA-treated groups (Figs. 5A–C). These observations were further confirmed by H&E staining, reinforcing the efficacy of UA, highlighting a reduction in alveolar atrophy in the meibomian glands and lacrimal glands following UA treatment, relative to the model group (Figs. 5D and E). To assess lipid secretion in the lacrimal glands, oil red O staining was performed, with staining intensity serving as an indicator of lipid obstruction. A darker staining intensity signified a more severe degree of blockage. The results showed significant blockage in both the meibomian and lacrimal glands in the model and vehicle groups, while no lipid accumulation was observed in the LC-UA and MC-UA groups (Figs. 5F and G). All the above results were statistically significant (Figs. 5H and I). These results are consistent with previous studies, which indicate that normal lacrimal glands contain minimal lipid content, while abnormal lipid deposition is a hallmark of lacrimal gland dysfunction [27]. This observation was further supported by LipidTOX staining, which confirmed UA's ability to reverse abnormal lipid deposition in both gland types (Fig. S10). Fluorescent staining of CD3^+^ cells revealed a statistically significant reduction in inflammatory infiltration of the lacrimal glands following UA treatment (Figs. 5J and K). These results highlight the multi-faceted therapeutic benefits of UA eye drops in addressing the complex pathophysiology of DE.

### 3.6 UA and DE target prediction

**Fig. 6:**
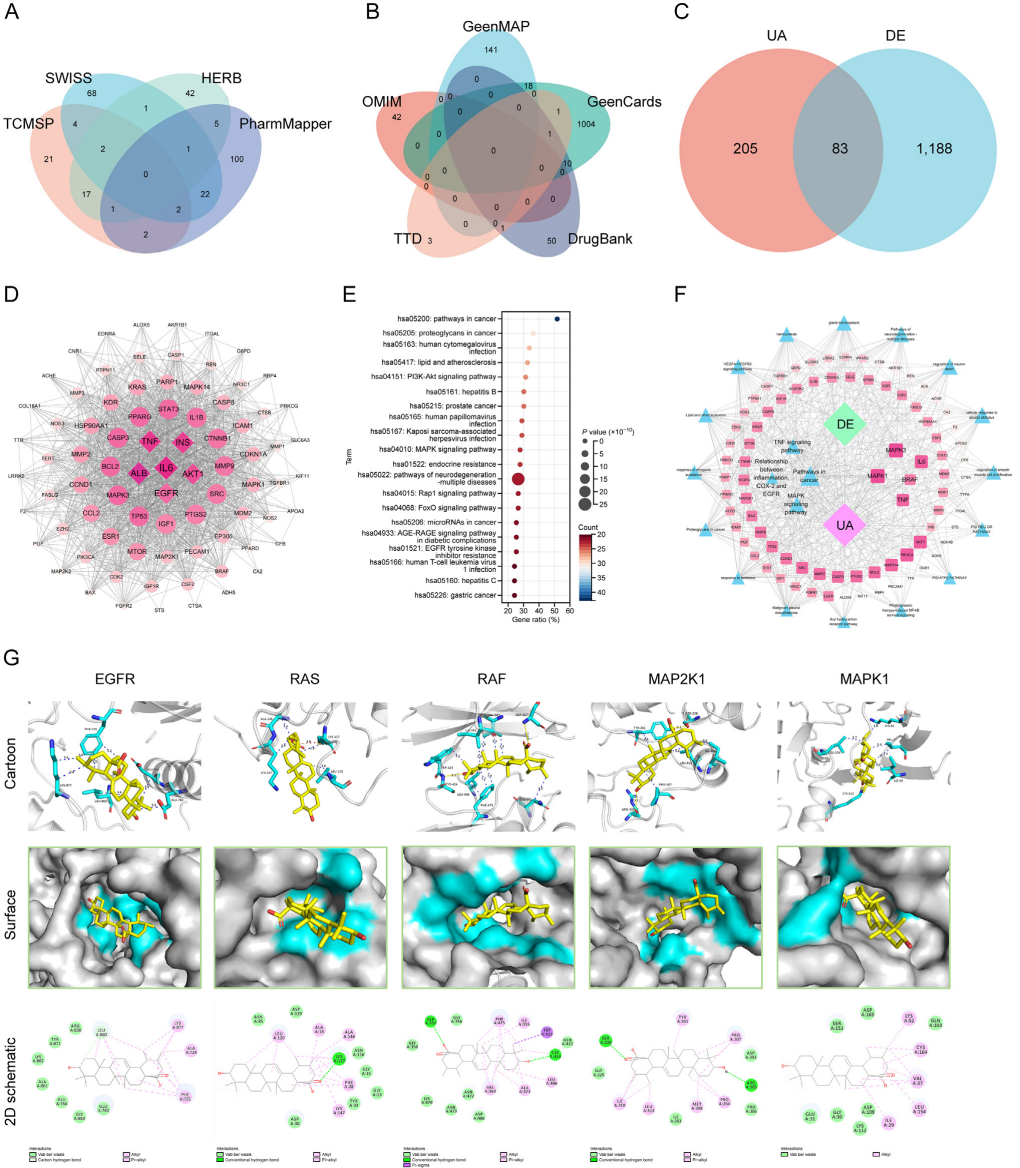
Ursolic acid (UA) and dry eye (DE) target prediction. (A) Screening targets for UA from various databases. (B) Screening targets for DE from different databases. (C) Venn diagram illustrating the intersecting targets between UA and DE. (D) Protein-protein interaction (PPI) network analysis to identify core targets. (E) Kyoto Encyclopedia of Genes and Genomes (KEGG) enrichment analysis using the Database for Annotation, Visualization and Integrated Discovery (DAVID) platform. (F) Drug-disease-target-pathway network analysis. (G) Molecular docking between UA and core therapeutic targets of DE. Cartoon mode, surface mode, and two-dimensional (2D) visualization of molecular docking are presented, with hydrogen bonding, hydrophobic interactions, and salt bridges represented in yellow, blue, and red, respectively. TCMSP: Traditional Chinese Medicine Systems Pharmacology Database and Analysis Platform; OMIM: Online Mendelian Inheritance in Man; TTD: Therapeutic Target Database; TNF: tumor necrosis factor; INS: insulin; IL-6: interleukin-6; ALB: albumin; AKT1: RAC-alpha serine/threonine-protein kinase; PI3K-Akt: phosphoinositide 3-kinase-protein kinase B; MAPK: mitogen-activated protein kinase; FoxO: forkhead box O; AGE-RAGE: advanced glycation endproducts-receptor for advanced glycation endproducts; EGFR: epidermal growth factor receptor; COX-2: cyclooxygenase-2; BRAF: v-raf murine sarcoma viral oncogene homolog B; RAS: rat sarcoma; RAF: rapidly accelerated fibrosarcoma; MAP2K1: MAPK kinase 1.

To further validate the therapeutic mechanism of UA in addressing DE syndrome, a network pharmacology framework was employed (Fig. S11). A comprehensive screening across four databases, TCMSP, SwissTargetPrediction, HERB, and PharmMapper, yielded a diverse set of 49 UA targets from TCMSP, 100 from SwissTargetPrediction, 69 from HERB, and 133 from PharmMapper (Fig. 6A and Supplementary data 1). Additionally, a thorough search of five other databases (TTD, OMIM, GeneCards, GeneMap, and DrugBank) identified 1,303 distinct targets associated with DE (Fig. 6B and Supplementary data 2), with specific distributions of 6 from TTD, 42 from OMIM, 1,034 from GeneCards, 159 from GeneMap, and 62 from DrugBank. After removing redundant entries, a Venn diagram revealed 83 common targets shared between UA and DE, indicating potential therapeutic intersections (Fig. 6C). To enhance the visualization and understanding of these intersecting targets, Cytoscape software was utilized to generate a clear depiction of the shared mechanisms (Fig. S12 and Supplementary data 3). To elucidate the underlying mechanisms of UA in treating DE, an in-depth network analysis, incorporating PPI networks, GO analysis, and KEGG enrichment analysis, was conducted. Core targets were imported into STRING database, and a stringent cross-linking score threshold of >0.4 was applied to construct the PPI network (Fig. S13A). Visualized using Cytoscape, the size and color intensity of the nodes correlated with the core coefficient of each target, emphasizing key cross-linking core targets such as EGFR, IL-6, TNF-α, and others as pivotal in the intersection of UA and DE mechanisms (Fig. 6D). GO functional annotations and KEGG pathway enrichments for the 83 shared targets were explored using the DAVID platform (Fig. 6E and Supplementary data 4). The top 20 MFs, CCs, and BPs related to these targets is shown in Figs. S13B–D. Further enrichment analysis conducted on the Metascape platform revealed 680 pathways, with the top 20 pathways being highlighted (Fig. S13E), including the EGFR and MAPK pathways. To integrate our findings and provide a comprehensive overview, the common targets, PPI data, and KEGG enrichment analysis results were imported into Cytoscape v.3.9.0, and a drug-disease-target-pathway network diagram was constructed (Fig. 6F). This network visually represents the intricate interplay between UA, DE, their shared targets, and the underlying biological pathways.

Molecular docking, a widely utilized technique, plays a pivotal role in elucidating the potential binding interactions between molecules. To deepen the understanding of UA's binding capability and interaction modes with core target proteins involved in DE, molecular docking studies were conducted using AutoDock and PyMOL software. Prior network pharmacological analyses suggested a potential link between UA and EGFR, yet the specific regulatory interplay between UA and EGFR in the context of DE remained unexplored. Consequently, EGFR, along with RAS, RAF, MAP2K1, and MAPK1, were selected as receptor targets, with UA as the ligand, to investigate the optimal binding configurations (Fig. 6G). The docking results provided comprehensive data on binding energies, interaction forces, and bond lengths (Table 2, Table 3). These metrics are essential for assessing binding affinity, as the absolute value of the binding energy correlates inversely with the dissociation force required, indicating stronger binding stability for higher absolute energy values. Notably, UA demonstrated the most favorable binding to RAF, with a binding energy of −9.3 kcal/mol, well below the −7.5 kcal/mol threshold observed for the other targets. Visualization of the docking sites revealed intricate binding interactions, supported by 2D interaction analyses that highlighted hydrogen bonding, hydrophobic interactions, and salt-bridge formations, particularly with MAPK1, as key modes of UA's engagement with these proteins. Crucial amino acid residues involved in these interactions included EGFR's PHE-725, MAP2K1's TYR-261, and RAF's TRP-423 and PHE-475, suggesting that UA may influence their functional dynamics. These results not only emphasize UA's capacity to stabilize its binding to targeted pathways but also underscore the specificity and strength of these interactions, with RAF leading the binding affinity, followed by EGFR, MAPK1, MAP2K1, and RAS. Based on these docking results, UA's therapeutic effects may be attributed to its modulation of the EGFR/RAS/RAF/MAP2K1/MAPK1 signaling pathway, offering a plausible mechanistic explanation for its potential role in treating DE.

**Table 2:** Details of molecular docking analysis of ursolic acid (UA) (PubChem CID: 64945).

Target	Uniprot ID	Binding energy (kcal/mol)
EGFR	Q01279	−8.9
RAS	P32883	−7.5
RAF	Q99N57	−9.3
MAP2K1/MEK1	P31938	−8.6
MAPK1/ERK2	P63085	−8.6

EGFR: epidermal growth factor receptor; RAS: rat sarcoma; RAF: rapidly accelerated fibrosarcoma; MAP2K1/MEK1: mitogen-activated protein kinase (MAPK) kinase 1; *ERK2*: extracellular signal regulated kinase 2.

**Table 3:** Details of molecular docking analysis.

Interaction	Binding energy (kcal/mol)	Hydrogen bonds	Hydrophobic interaction	Salt bridges
UA-EGFR	−8.9	−	PHE-725A (3.45 Å,3.29 Å, and 3.73 Å), GLU-760A (3.86 Å), GLU-764A (3.59 Å), LEU-860A (3.58 Å), and LYS-877A (3.82 Å and 3.58 Å)	−
UA-MAPK1	−8.6	−	ILE-29A (3.35 Å), LYS-52A (3.80 Å), LEU-154A (3.69 Å), and VAL-37A (3.43 Å and 3.71 Å)	LYS-112A (4.47 Å)
UA-MAP2K1	−8.6	SER-228A (3.08 Å) and ARG-305A (3.15 Å)	TYR-261A (3.82 Å), PRO-307A (3.88 Å), LEU-313A (3.40 Å), and ILE-320A (3.35 Å)	−
UA-RAF	−9.3	SER-357A (3.19 Å) and CYS-424A (3.33 Å)	VAL-363A (3.36 Å,3.36 Å, and 2.59 Å), ILE-355A (3.83 Å), TRP-423A (3.20 Å), LEU-406A (3.40 Å), PHE-475A (3.36 Å, 3.55 Å, and 3.21 Å), and ASN-472A (3.98 Å)	−
UA-RAS	−7.5	LYS-117A (3.60 Å)	LYS-117A (3.76 Å), LEU-120A (3.60 Å), ALA-146A (3.41 Å), and LYS-147A (3.83 Å)	−

−: no data. UA: ursolic acid; EGFR: epidermal growth factor receptor; MAPK1: mitogen-activated protein kinase 1; MAP2K1: MAPK kinase 1; RAF: rapidly accelerated fibrosarcoma; RAS: rat sarcoma.

### 3.7 Single cell sequencing database analysis

**Fig. 7:**
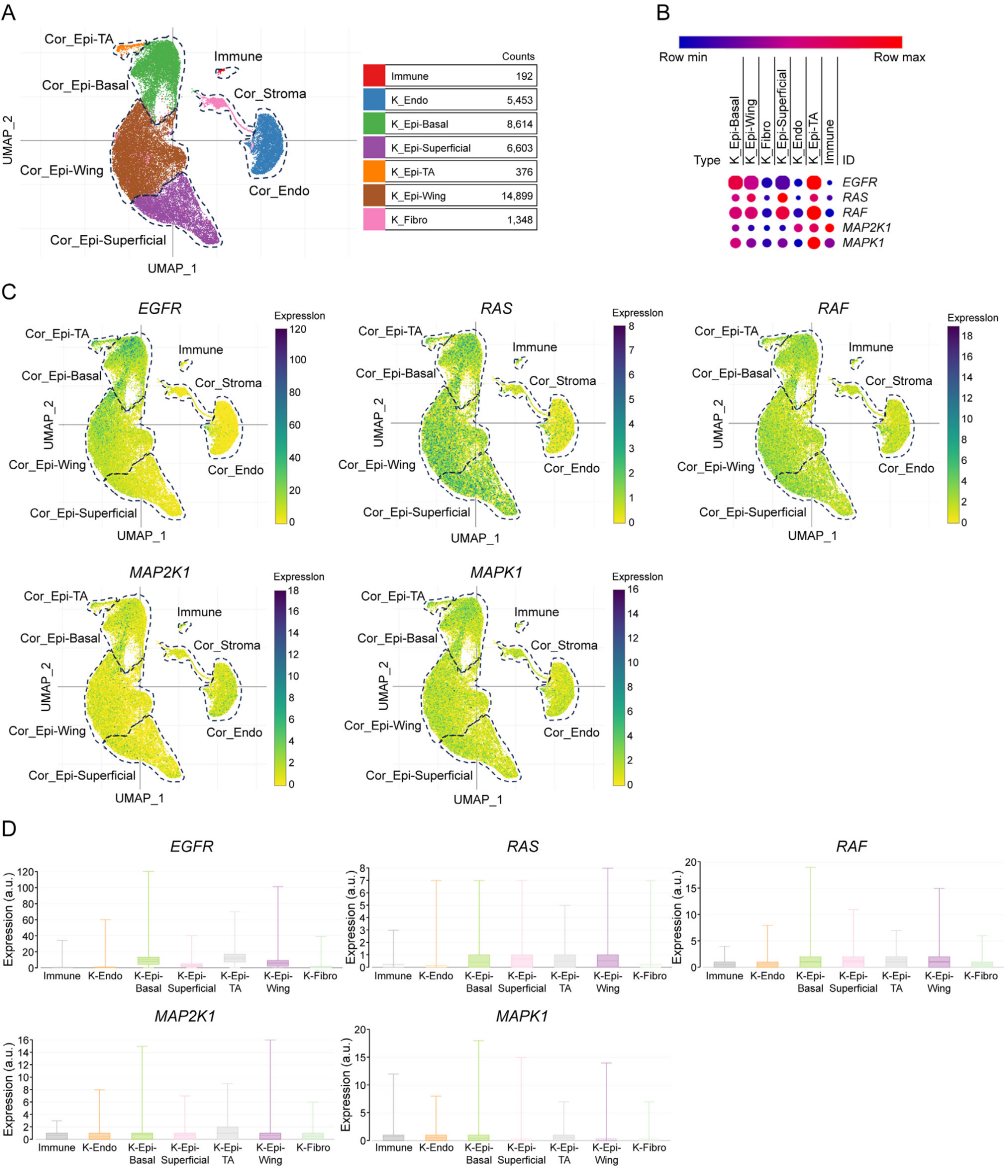
Single-cell sequencing database analysis. (A) Distribution of cell clusters in the cornea. (B) *Comparison of epidermal growth factor receptor* (*EGFR*), *rat sarcoma* (*RAS*), *rapidly accelerated fibrosarcoma* (*RAF*), *mitogen-activatedprotein kinase* (*MAPK*) *kinase 1* (*MAP2K1*), and *MAPK1* in the cornea. (C) Spatial distribution of *EGFR*, *RAS*, *RAF*, *MAP2K1*, and *MAPK1* within the cornea. (D) Quantitative expression levels of *EGFR*, *RAS*, *RAF*, *MAP2K1*, and *MAPK1* in the cornea. UMAP: uniform manifold approximation and projection; Cor: cornea; Epi: epithelium; Endo: endothelium; TA: transit amplify; Fibro: fibroblast.

To elucidate the mechanisms by which UA modulates DE following its interaction with the anterior eye segment, a single-cell histological analysis was performed using an extensive database. A detailed depiction of the cellular architecture of the anterior eye segment was shown in Fig. S14A, while the distribution and expression patterns of EGFR, RAS, RAF, MAP2K1, and MAPK1 within this region was shown (Figs. S14B, S14C, and S15). Consistent with prior research, the analysis confirms that EGFR is predominantly localized to the surface of epithelial cells, particularly in the corneal and conjunctival epithelium, as revealed by the single-cell histological data [28,29]. Notably, RAS demonstrates a dual localization, being not only abundant in the corneal and conjunctival epithelium but also widely distributed among cup cells, emphasizing its pivotal functional role. Further investigation of EGFR expression within corneal clusters identifies its concentrated presence in the superficial epithelium, pterygoid epithelium, and basal cell layers (Fig. 7A). In contrast, *RAS*, *RAF*, *MAP2K1*, and *MAPK1* exhibit a relatively uniform distribution across the cornea (Figs. 7B–D), underscoring the complex interplay of these signaling molecules in maintaining ocular health. These results provide critical insights into the mechanisms by which UA may alleviate DE through its modulation of these key signaling pathways.

### 3.8 RNA sequencing analysis

**Fig. 8:**
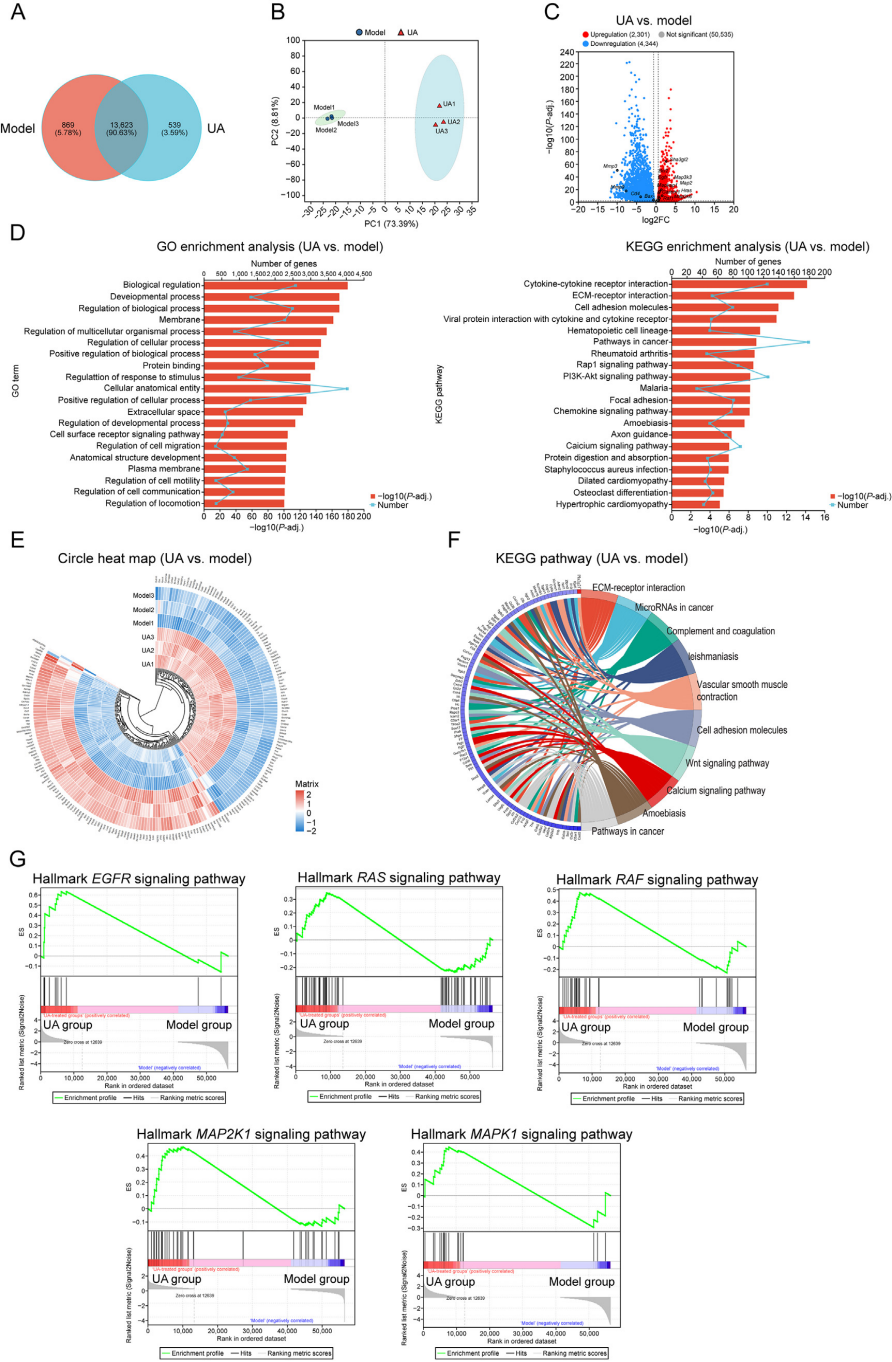
RNA sequencing analysis of the cornea treated with ursolic acid (UA). (A) Venn diagram illustrating differences in gene numbers between the UA and model groups. (B) Principal component analysis (PCA) depicting transcriptional differences between the UA and model groups. (C) Volcano plot showing differentially expressed genes. (D) Gene Ontology (GO) analysis (left) and Kyoto Encyclopedia of Genes and Genomes (KEGG) pathway enrichment analysis (right) comparing the UA and model groups. (E) Heatmap displaying gene expression levels across all samples. (F) Search Tool for the Retrieval of Interacting Genes (STRING) diagram visualizing enriched gene functions and pathways from the KEGG database between the UA and model groups. (G) Gene set enrichment analysis (GSEA) diagram illustrating the enrichment of epidermal growth factor receptor (*EGFR*), rat sarcoma (*RAS*), rapidly accelerated fibrosarcoma (*RAF*), mitogen-activated protein kinase (*MAPK*) kinase 1 (*MAP2K1*), and *MAPK1*. PC: principal component; FC: fold change; ECM: extra cellular matrix; PI3K-Akt: phosphoinositide 3-kinase-protein kinase B; ES: enrichment score.

To further investigate the mechanisms by which UA exerts its effects on DE, RNA sequencing was conducted on corneal samples from the UA treatment group and the model group. The analysis revealed differences in gene expression, with 13,623 genes co-expressed between the two groups, representing 90.63% of the total genes identified (Fig. 8A). Principal component analysis (PCA) demonstrated distinct clustering of the UA and model group, indicating independent transcriptional profiles (Fig. 8B). Differential expression analysis identified 2,301 upregulated and 4,344 downregulated genes in the UA group compared to the model group. Notably, genes such as *EGFR*, *RAS*, *RAF*, *MAPK1*, *MAP2K1*, and *Bcl2* exhibited upregulation, while *CD4*^*+*^, *MMP-3*, and *BAX* were significantly downregulated (Fig. 8C). These results suggest that UA may positively regulate the EGFR signaling pathway and attenuate corneal apoptosis during DE. GO and KEGG pathway enrichment analyses highlighted the enriched functions and pathways of differentially expressed genes (Fig. 8D). A heatmap depicting gene expression profiles between the UA and model group further illustrated these differences (Fig. 8E). Visualized through string diagrams, the enriched gene sets included those associated with ocular surface cellular processes, developmental processes, and biological regulation (Fig. S16). These results suggest that UA alleviates DE by promoting cellular proliferation and inhibiting cell death. Additionally, DE was characterized by significant activation of immunity-related processes, including cytokine-cytokine receptor interactions (Fig. 8F). Gene set enrichment analysis (GSEA) further identified distinct expression trends among key gene sets. Notably, genes such as *EGFR* were significantly positively enriched in the UA group, with a higher enrichment score (ES) peak for *EGFR* relative to other gene sets. This indicates that UA modulates the EGFR/RAS/RAF/MAP2K1/MAPK1 pathway as a central mechanism in mitigating DE (Fig. 8G).

### 3.9 Mechanism validation of UA for DE

**Fig. 9:**
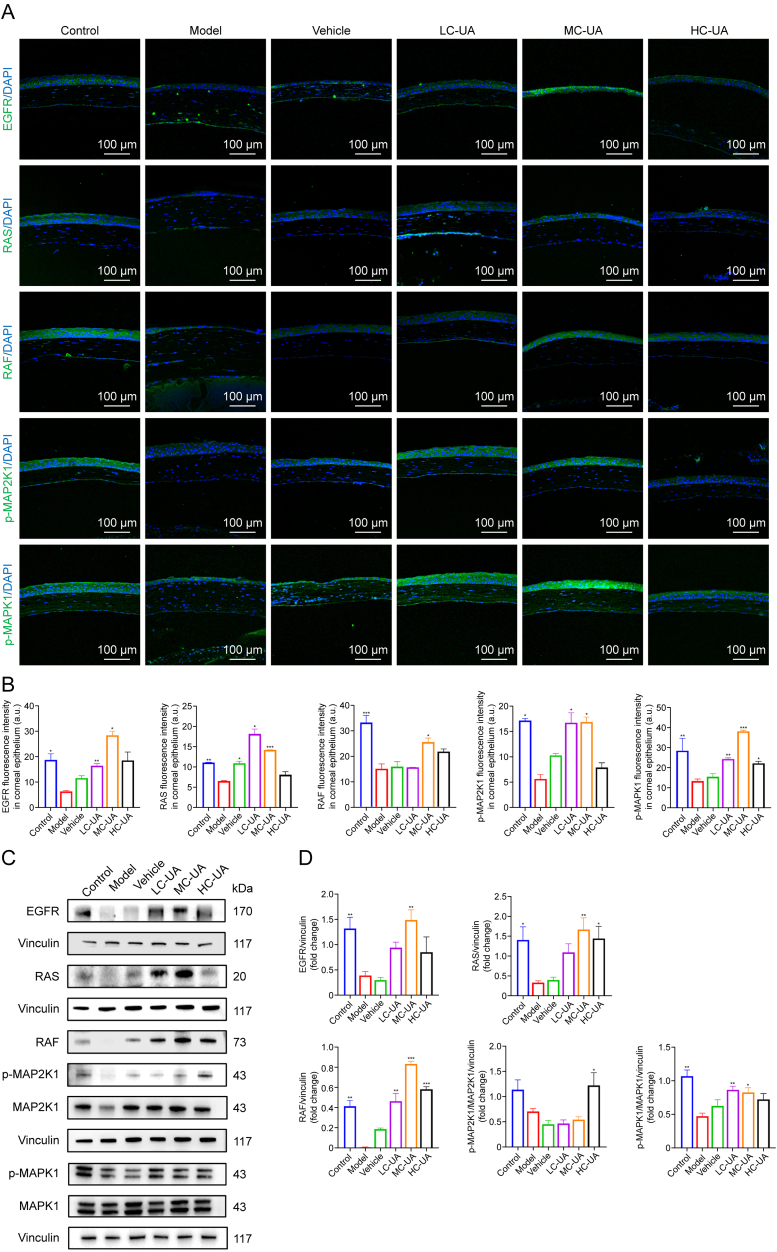
Ursolic acid (UA) alleviates dry eye (DE) via epidermal growth factor receptor (EGFR)/rat sarcoma (RAS)/rapidly accelerated fibrosarcoma (RAF)/mitogen-activated protein kinase (MAPK) kinase 1 (MAP2K1)/MAPK1 signaling pathway. (A) Immunofluorescence staining of EGFR, RAS, RAF, p-MAP2K1, and p-MAPK1 in the cornea. (B) Quantification of immunofluorescence intensity for EGFR, RAS, RAF, p-MAP2K1, and p-MAPK1 in the cornea (*n* = 3–4 per group). (C) Western blot analysis of EGFR, RAS, RAF, p-MAP2K1, MAP2K1, p-MAPK1, and MAPK1 in the cornea. (D) Quantification of EGFR, RAS, RAF, p-MAP2K1/MAP2K1, and p-MAPK1/MAPK1 protein levels in the cornea (*n* = 4–7 per group). Data are presented as mean ± standard error of the mean (SEM). ^∗^*P* < 0.05, ^∗∗^*P* < 0.01, and ^∗∗∗^*P* < 0.001, comparison with the model group. DAPI: 4′,6-diamidino-2-phenylindole; LC-UA: low concentration of UA; MC-UA: medium concentration of UA; HC-UA: high concentration of UA.

**Fig. 10:**
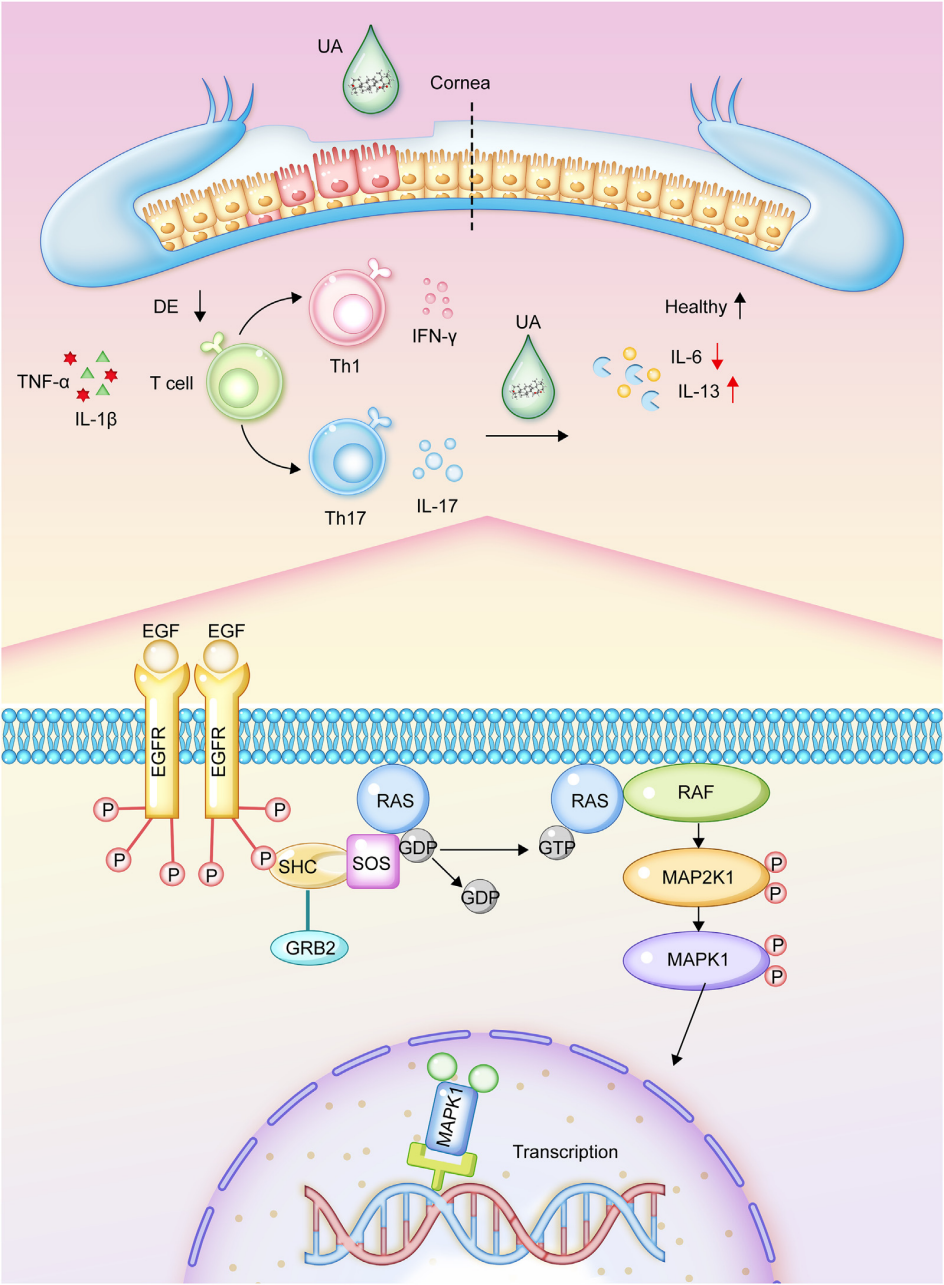
Schematic diagram illustrating the mechanism of ursolic acid (UA) in treating dry eye (DE). TNF-α: tumor necrosis factor alpha; IL-1β: interleukin-1 beta; Th1: T helper 1 cell; IFN-γ: interferon gamma; EGF: epidermal growth factor; EGFR: EGF receptor; SHC: Src homology 2 domain containing protein; GRB2: growth factor receptor-bound protein 2; SOS: Son of Sevenless; RAS: rat sarcoma; GDP: guanosine diphosphate; GTP: guanosine triphosphate; RAF: rapaidly accelerated fobrosacrom; MAP2K1: mitogen-activated protein kinase (MAPK) kinase 1.

To validate the findings derived from network pharmacology, molecular docking analyses, and single-cell sequencing, molecular biology experiments utilizing immunofluorescence techniques were conducted to quantify the expression levels and localization of key proteins within the EGFR/RAS/RAF/MAP2K1/MAPK1 signaling cascade in the cornea. The experimental results demonstrated significant increases in the fluorescence intensities of EGFR, RAS, RAF, p-MAP2K1, and p-MAPK1 in corneal tissues treated with UA eye drops, compared to the model group (Figs. 9A and B). These observations were further substantiated by Western blot analysis, which revealed a marked reduction in the phosphorylation levels of MAP2K1 and MAPK1 in the corneal cytoplasm of the model group compared to the control group. In contrast, treatment with UA eye drops significantly enhanced the phosphorylation of MAP2K1 and MAPK1 (Figs. 9C and D). Additionally, the expression profiles of RAS, RAF, and EGFR proteins in the model group paralleled the trends observed for p-MAP2K1 and p-MAPK1, suggesting that DE inhibits the EGFR/RAS/RAF/MAP2K1/MAPK1 signaling pathway. Conversely, UA eye drop administration effectively alleviated DE by activating this pathway. These results underscore the therapeutic potential of UA in modulating the EGFR/RAS/RAF/MAP2K1/MAPK1 signaling cascade for the treatment of DE. Single-cell histological data revealed the presence of diverse cell types in the cornea, including epithelial cells, stromal cells, fibroblasts, and immune cells. Notably, EGFR, RAS, RAF, MAP2K1, and MAPK1 were predominantly localized in corneal epithelial cells. To investigate whether UA primarily targets the corneal epithelium, hypertonicity-induced HCEs were used for further validation. Consistent with previous findings, EGFR, RAS, RAF, MAP2K1, and MAPK1 were significantly upregulated in HCEs treated with UA compared to the HS group (Fig. S17). These cellular-level results further elucidate the mechanism by which UA modulates the EGFR/RAS/RAF/MAP2K1/MAPK1 signaling pathway, reinforcing its potential as a therapeutic agent for DE (Fig. 10). Collectively, these observations refine the understanding of UA's action mechanism and highlight its promise as a potential treatment for DE.

## 4 Discussion

DE, a prevalent and multifaceted ocular surface disorder, affects a significant portion of the global population [30]. The significance of this research in the pursuit of safer and more effective treatments for DE cannot be overstated. This study explored the therapeutic effects of UA on ocular surface dysfunction in DE and further investigated its potential mechanisms of action through both *in vivo* and *in vitro* experiments.

UA is a noteworthy extract, well-known for its potent anti-inflammatory properties [31,32]. Based on this, it was hypothesized that UA eye drops may have therapeutic effects on DE. To better simulate mixed DE, a combination of scopolamine and BAC was used. Scopolamine disrupts tear production by inhibiting parasympathetic nerve activity, leading to a reduction in both tear quantity and quality, accompanied by inflammatory cell infiltration [33]. As expected, treatment with UA eye drops resulted in increased tear secretion and a reduction in lacrimal inflammatory cell infiltration in mice with DE. In this study, the lacrimal and meibomian glands of the model group showed abnormal lipid deposition, likely attributed to BAC-induced DE. BAC, a common preservative in ophthalmic medications, exerts toxic effects on the epithelial cells of the meibomian glands, causing glandular atrophy and cell death [34]. This leads to impaired meibomian gland opening and obstruction, hindering lipid drainage and resulting in lipid accumulation within the gland lumen. Notably, our findings suggest that UA eye drops may not only stimulate tear production but also mitigate abnormal lipid deposition in the lacrimal and meibomian glands. These results highlight the promising therapeutic potential of UA for addressing DE and underscore the need for further exploration in this promising field. Additionally, network pharmacology results suggest that UA's ability to alleviate DE symptoms may involve modulation of lipid metabolism.

In this study, a comprehensive “disease-gene-target-drug” network framework was developed to predict drug mechanisms in a cost-effective and streamlined manner. The therapeutic mechanisms of UA were further validated at both molecular docking and molecular biology levels. These findings demonstrate that UA eye drops exert their therapeutic effects by activating the EGFR/RAS/RAF/MAP2K1/MAPK1 signaling pathway, positioning UA as a promising candidate for the treatment of DE with significant research and clinical implications.

EGFR is critical for regulating cell proliferation, differentiation, and apoptosis, primarily resides on the cell surface as a monomer with minimal kinase activity. Upon binding to its ligand, conformational changes occur within the tyrosine kinase domain of EGFR, leading to autophosphorylation at the adenosine triphosphate (ATP)-binding site. This activation triggers a cascade of intracellular signaling pathways, including phosphoinositide 3-kinase (PI3K)/protein kinase B (Akt), RAS/RAF/MAP2K1/MAPK1, and Janus kinase (JAK)/signal transducer and activator of transcription (STAT), ultimately engaging downstream effectors and orchestrating diverse cellular responses [28,35,36]. Within the RAS/RAF/MAP2K1/MAPK1 signaling axis, EGFR activation promotes cell growth and proliferation while inhibiting apoptosis, playing a critical role in maintaining cellular homeostasis [29]. These findings align with the transcriptome sequencing results presented in this study, which indicate that UA contributes to processes such as cell division, growth, and development in alleviating DE. This evidence underscores the theoretical foundation for UA's ability to improve DE symptoms via the EGFR/RAS/RAF/MAP2K1/MAPK1 pathway, further establishing the connection between DE and EGFR. Moreover, this study provides additional support for the role of EGFR in corneal epithelial repair and offers a new perspective on the pathophysiology of DE. The transcriptome results also suggested that UA promotes axonal growth, linking this effect to processes such as oocyte meiosis and retinol metabolism. This finding implies that UA may enhance dark adaptation through retinol metabolism, thereby reducing visual fatigue and alleviating DE symptoms. Wang et al. [37] demonstrated that maternal oral administration of UA enhanced neuroprotection by upregulating transcription of the intestinal acidic ceramidase-1 (*Asah-1*) gene via sphingosine-1-phosphate, thereby mitigating developmental axonal growth defects in offspring. This suggests that UA has potential applications in ophthalmology not only through topical eye drops for ocular surface diseases but also through oral administration to promote neural axon regeneration. However, further research is required to determine the specific axonal pathways affected.

This study demonstrated that UA eye drops effectively reduce the expression of cleaved caspase-8, inhibit apoptosis, and suppress inflammation. This dual action, targeting both apoptosis and inflammation, is likely pivotal in disrupting the vicious cycle underlying DE and alleviating its symptoms. However, the precise mechanistic link between UA-mediated inhibition of apoptosis and inflammation warrants further investigation in future research on UA. The activity of caspase-8 is essential for the activation of the NF-κB signaling pathway, which in turn triggers the secretion of cytokines [38]. NF-κB serves as a key mediator in the progression of DE, frequently being activated during the disease process [39]. This activation promotes the release of various inflammatory cytokines, thereby intensifying the inflammatory response. Furthermore, research has demonstrated that TNF-α, a potent cytokine, can enhance the apoptotic process by activating the caspase-8 pathway. This highlights the complex interaction between inflammation and apoptosis in the pathogenesis of DE [40]. This reciprocal relationship creates a self-perpetuating cycle that exacerbates disease severity. UA has gained attention for its potential to modulate this pathological cascade. Notably, UA has been reported to downregulate NF-κB activity, offering a promising therapeutic strategy for DE [41]. This study further corroborates these findings, showing that UA reduces p-NF-κB expression (Fig. S18). It is plausible that the ability of UA eye drops to inhibit apoptosis and inflammation can be partially attributed to their modulation of cleaved caspase-8 expression via activation of the EGFR/RAS/RAF/MAP2K1/MAPK1 signaling pathway. This pathway may indirectly mitigate NF-κB activation and reduce the secretion of inflammatory cytokines. However, this hypothesis is based on current findings and remains speculative. The exact mechanisms through which UA eye drops simultaneously inhibit apoptosis and inflammation remain unclear and present a valuable avenue for future research. Further studies are required to elucidate the molecular pathways involved and to confirm the therapeutic potential of UA in this context.

The onset of DE lesions is characterized by the activation of the NF-κB signaling pathway, driving the ocular surface into a heightened inflammatory state. UA has been shown to inhibit this pathway, thereby reducing the secretion of key inflammatory mediators, including IL-1β, TNF-α, IL-17, and IFN-γ. Under inflammatory conditions, CD4^+^ T cells are activated and differentiate into IFN-γ-producing CD4^+^ T helper 1 (Th1) cells and IL-17-secreting CD4^+^ Th17 cells. These effector cells further amplify the inflammatory response, perpetuating a self-sustaining cycle within the conjunctiva and lacrimal glands. This cycle compromises corneal barrier integrity and reduces overall cellular health, underscoring the critical interplay between immune activation and tissue damage in DE pathogenesis. These insights highlight the urgent need for targeted therapeutic approaches to disrupt this destructive cycle [42]. Research has demonstrated that IL-1β and TNF-α upregulate MMPs, while IL-17 specifically enhances MMP-3 expression, thereby disrupting corneal tight junction integrity [25]. Furthermore, IL-17 contributes to the formation of corneal lymphatic vessels, providing a pathway for antigen-presenting cells to access lymphatic circulation [3]. These findings align with the results of this study, suggesting that UA enhances corneal epithelial barrier function by inhibiting IL-17-producing CD4^+^ Th17 cells. Additionally, IFN-γ plays a pivotal role in regulating cellular functions, including mucin expression, which is essential for maintaining ocular surface health. Our findings indicate that UA suppresses IFN-γ secretion from CD4^+^ Th1 cells, mitigating cellular depletion and contributing to the stabilization of tear film rupture time in patients with DE [43,44]. Based on these observations, it is posited that UA exerts its therapeutic effects on DE by attenuating immune overactivation at the ocular surface. Specifically, UA inhibits the differentiation of CD4^+^ T cells into the pro-inflammatory CD4^+^ Th1 and CD4^+^ Th17 subsets, subsequently reducing the expression of inflammatory mediators such as IFN-γ and IL-17. This dual action enhances corneal barrier function, alleviates conjunctival inflammation, increases conjunctival cell density, and ultimately provides effective relief from DE symptoms.

UA emerges as a promising natural anti-inflammatory agent, though its limited solubility remains a significant challenge. Despite advancements in this study, including moderate improvements in UA's solubility through innovative techniques such as ultrasound and heating, the solubility remains suboptimal, potentially influencing drug absorption and efficacy. The concentration used in this study represents an optimized balance between solubility and therapeutic efficacy within current constraints. Future strategies to enhance UA's performance include targeted structural modifications, particularly at specific carbon positions within the UA molecule, to improve solubility and activity. Moreover, nanotechnology-based drug delivery systems offer tremendous potential to enhance UA's bioavailability, selectivity, and dosage precision, thereby unlocking new therapeutic possibilities. Furthermore, the specificity of UA's activation of EGFR and its comparative effects relative to EGF warrant further exploration to refine its therapeutic profile.

In summary, this study underscores the exceptional biological safety and efficacy of UA, both *in vitro* and *in vivo*, solidifying its potential as a therapeutic agent for inflammatory ocular surface conditions, particularly DE. UA's demonstrated ability to effectively repair corneal epithelial damage and alleviate ocular surface injuries associated with DE highlights its significant promise in ophthalmic medicine. Additionally, this work provides a new perspective on DE treatment, opening pathways for further research and clinical applications.

## 5 Conclusion

For the first time, this study delineates the protective effects of UA against DE and elucidates the precise mechanisms underlying its therapeutic actions. UA exerts its effects by modulating the EGFR/RAS/RAF/MAP2K1/MAPK1 signaling pathway, leading to the repair of corneal epithelial barrier function, enhancement of tear secretion, and suppression of ocular surface inflammation and damage. These findings position UA as a compelling candidate for the development of clinical pharmacological therapies for DE.

## CRediT authorship contribution statement

**Qinghe Zhang:** Writing – review & editing, Writing – original draft, Validation, Software, Investigation, Formal analysis, Data curation. **Ke Yan:** Methodology, Investigation, Formal analysis, Data curation. **Yufei Lv:** Writing – review & editing Methodology. **Qiuping Liu:** Writing – review & editing, Supervision, Resources, Project administration, Methodology, Conceptualization. **Yi Han:** Writing – review & editing, Visualization, Supervision, Formal analysis, Conceptualization. **Zuguo Liu:** Writing – review & editing, Supervision, Resources, Project administration, Funding acquisition.

## Declaration of competing interest

The authors declare that there are no conflicts of interest.
