# Supplementary material for: Ursolic acid ameliorates ocular surface dysfunction in dry eye via targeting EGFR/RAS/RAF/MAP2K1/MAPK1 pathway
Source: J Pharm Anal. 2025 Apr 3;15(11):101294. doi: 10.1016/j.jpha.2025.101294 (PMC12702019; doi:10.1016/j.jpha.2025.101294)
Supplement: Multimedia component 1 [file mmc1.zip › JPHA_101294 Supplementary Data/Supplementary Figures.docx]

**Supplementary Information**

` `
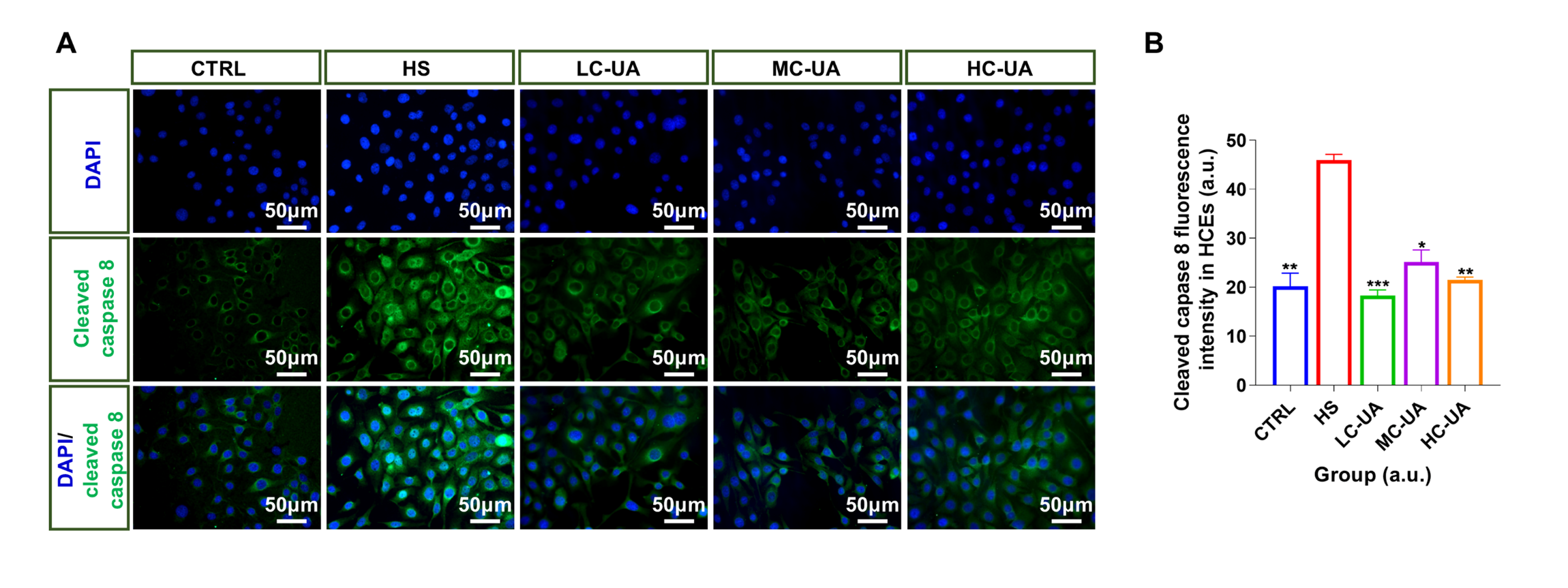


**Fig.** **S1. Ursolic acid (UA) effectively salvages the apoptosis of HCEs subjected to hypertonic stress (HS).** (**A**) Representative images of Cleaved Caspase 8 in HCEs after incubation with different concentrations of UA in 500 mOsM medium for 24 h. (**B**) Fluorescence intensity quantification of Cleaved Caspase 8 in HCEs after incubation with different concentrations of UA in 500 mOsM medium for 24 h. (*n* = 3 per group). Data are expressed as mean ± Standard Error of the Mean (S.E.M.). *P < 0.05, **P < 0.01, and ***P < 0.001, comparison between the specified group and the HS group. CTRL: control groups; HS: hypertonic stress; LC-UA: low concentration of UA; MC-UA: medium concentration of UA; HC-UA: high concentration of UA; DAPI: 4',6-diamidino-2-phenylindole.


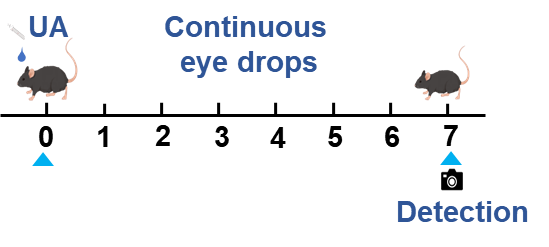


**Fig. S2. Ursolic acid (UA) presents biosecurity when applies *in vivo*.** Schematic diagram of safety test *in vivo*.


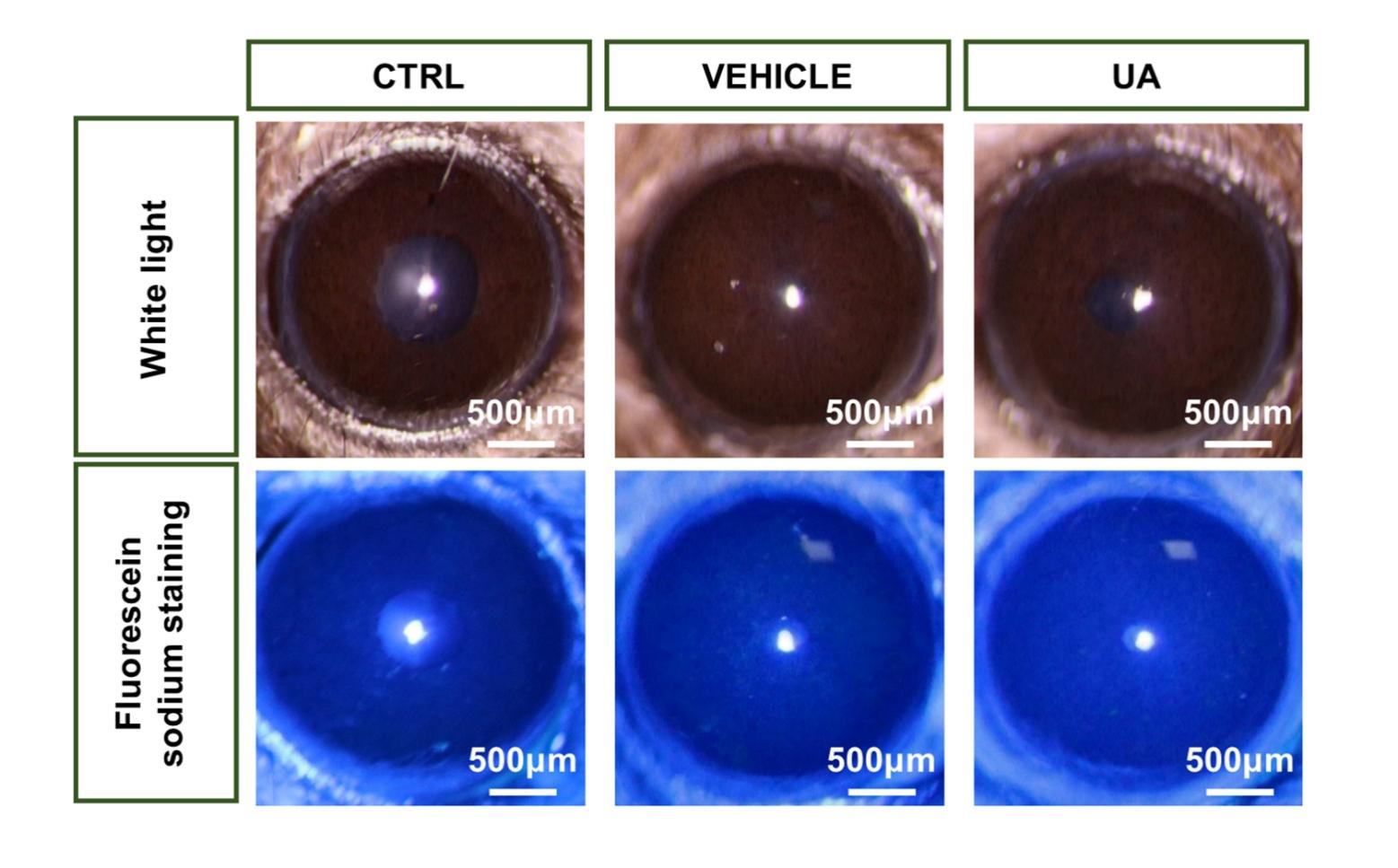


**Fig. S3. Ursolic acid (UA) presents biosecurity when applies *in vivo*.** Corneal fluorescence staining under slit lamp.CTRL: control groups; VEHICLE: vehicle groups.


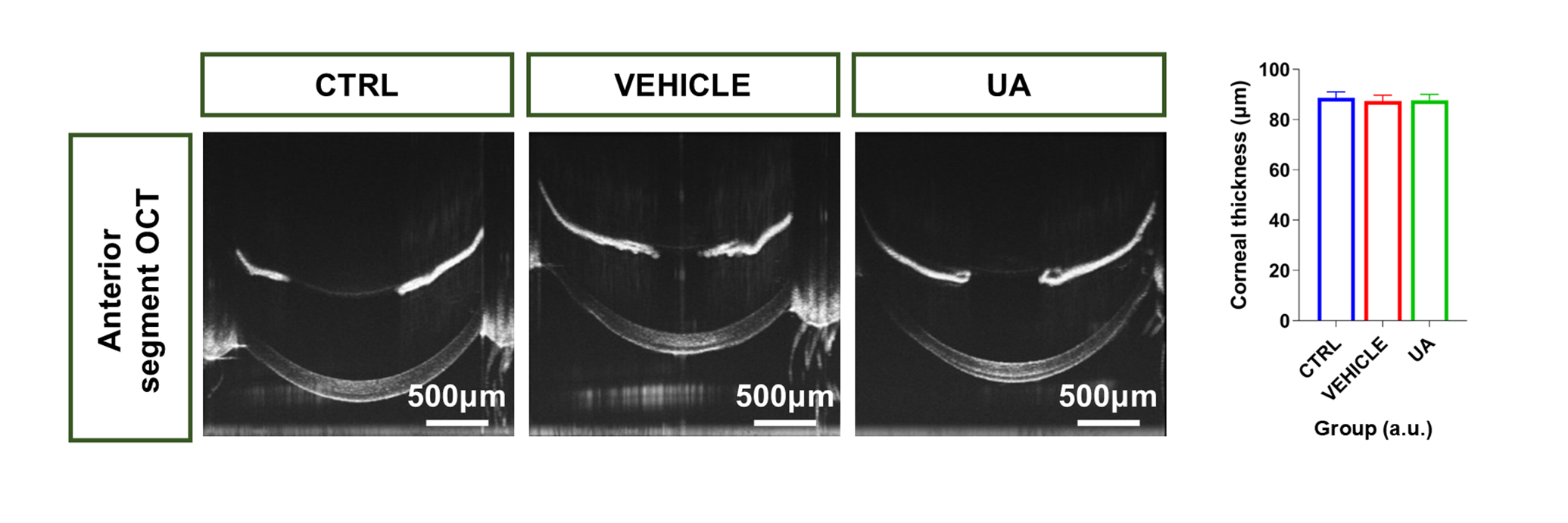


**Fig. S4. Ursolic acid (UA) presents biosecurity when applies *in vivo*.** Anterior segment optical coherence tomography (OCT) images and statistical analysis of corneal thickness (*n* = 3 per group). Data are expressed as mean ± Standard Error of the Mean (SEM).CTRL: control groups; VEHICLE: vehicle groups.


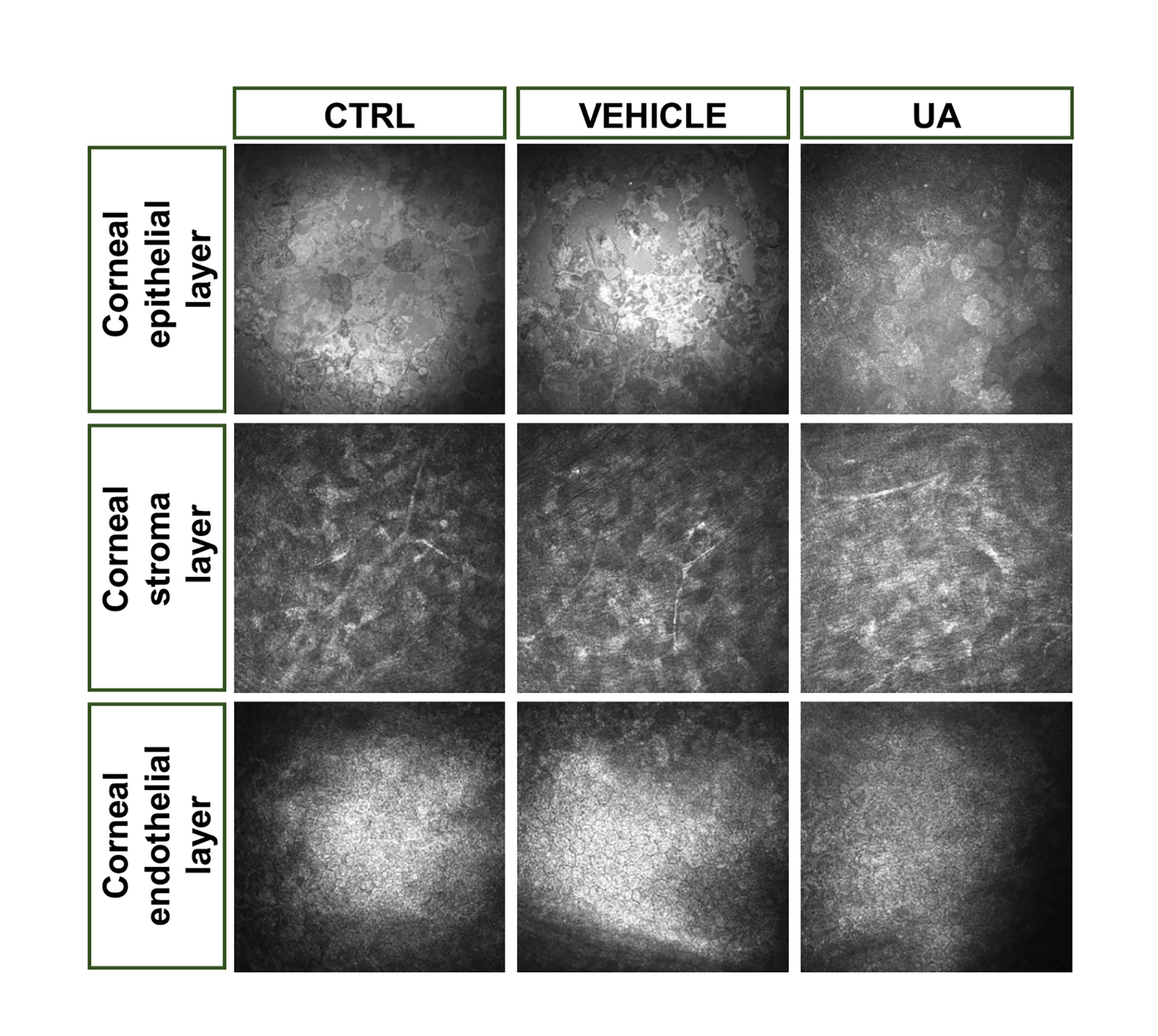


**Fig. S5. Ursolic acid (UA) presents biosecurity when applies *in vivo*.** Representative in vivo confocal laser corneal microscopy images.CTRL: control groups; VEHICLE: vehicle groups.


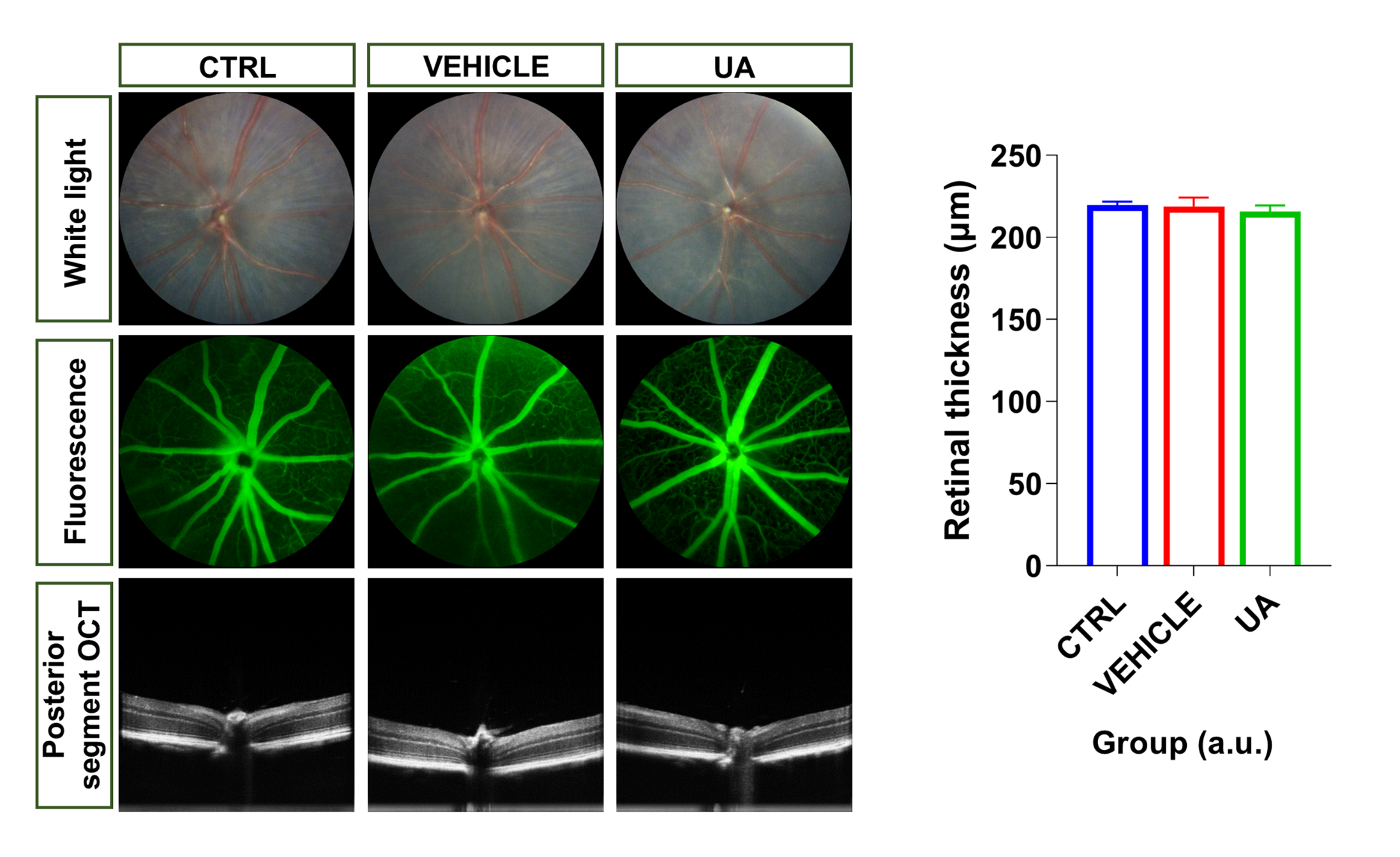


**Fig. S6. Ursolic acid (UA) presents biosecurity when applies *in vivo*.** Representative diagrams of fundus photography, fundus fluorescein angiography and posterior segment optical coherence tomography (OCT). And statistical analysis of retinal thickness (*n* = 4 per group). Data are expressed as mean ± Standard Error of the Mean (SEM).CTRL: control groups; VEHICLE: vehicle groups.


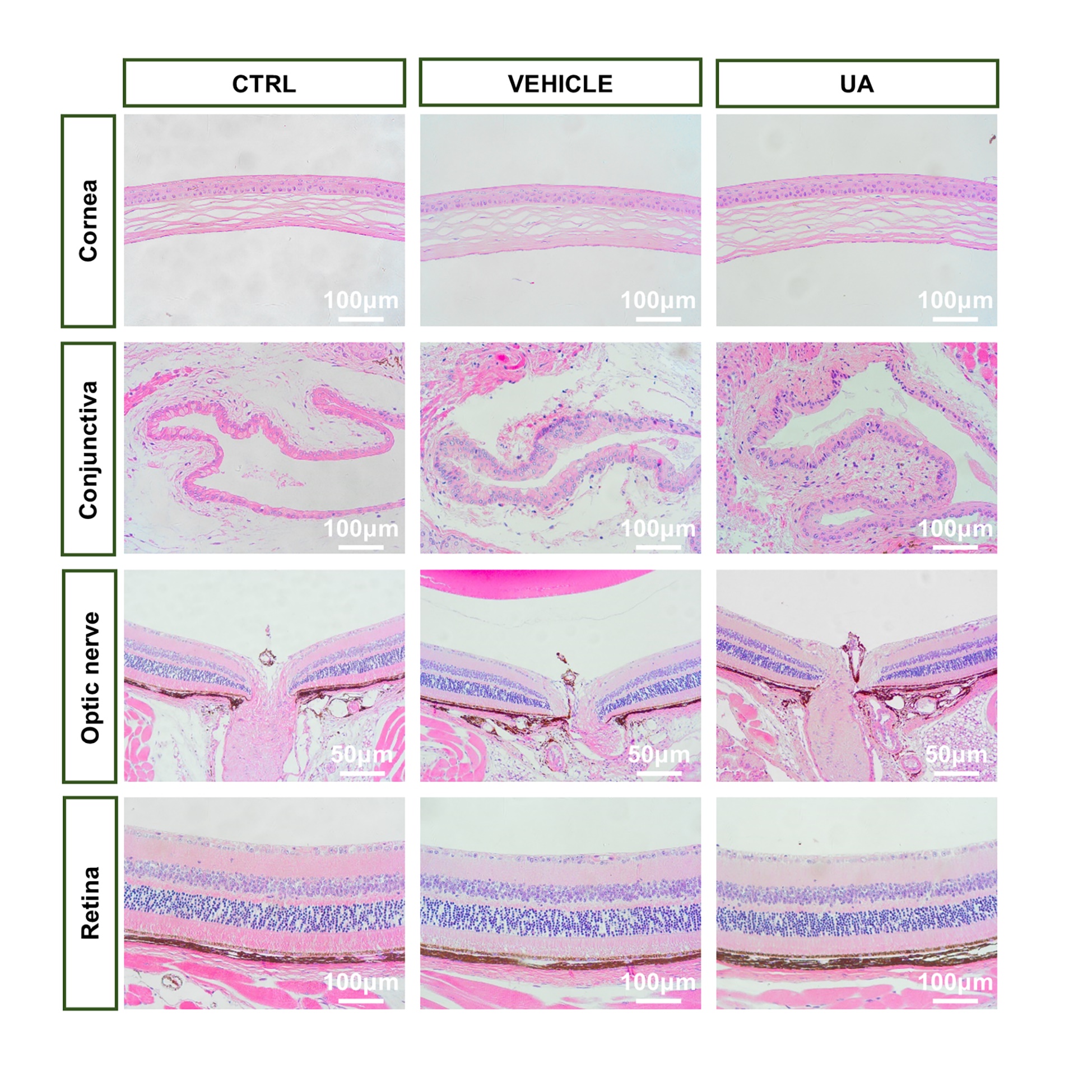


**Fig. S7. Ursolic acid (UA) presents biosecurity when applies *in vivo*.** Representative diagram of HE staining of corneal sections, conjunctiva sections, optic nerve sections and retina sections.CTRL: control groups; VEHICLE: vehicle groups.


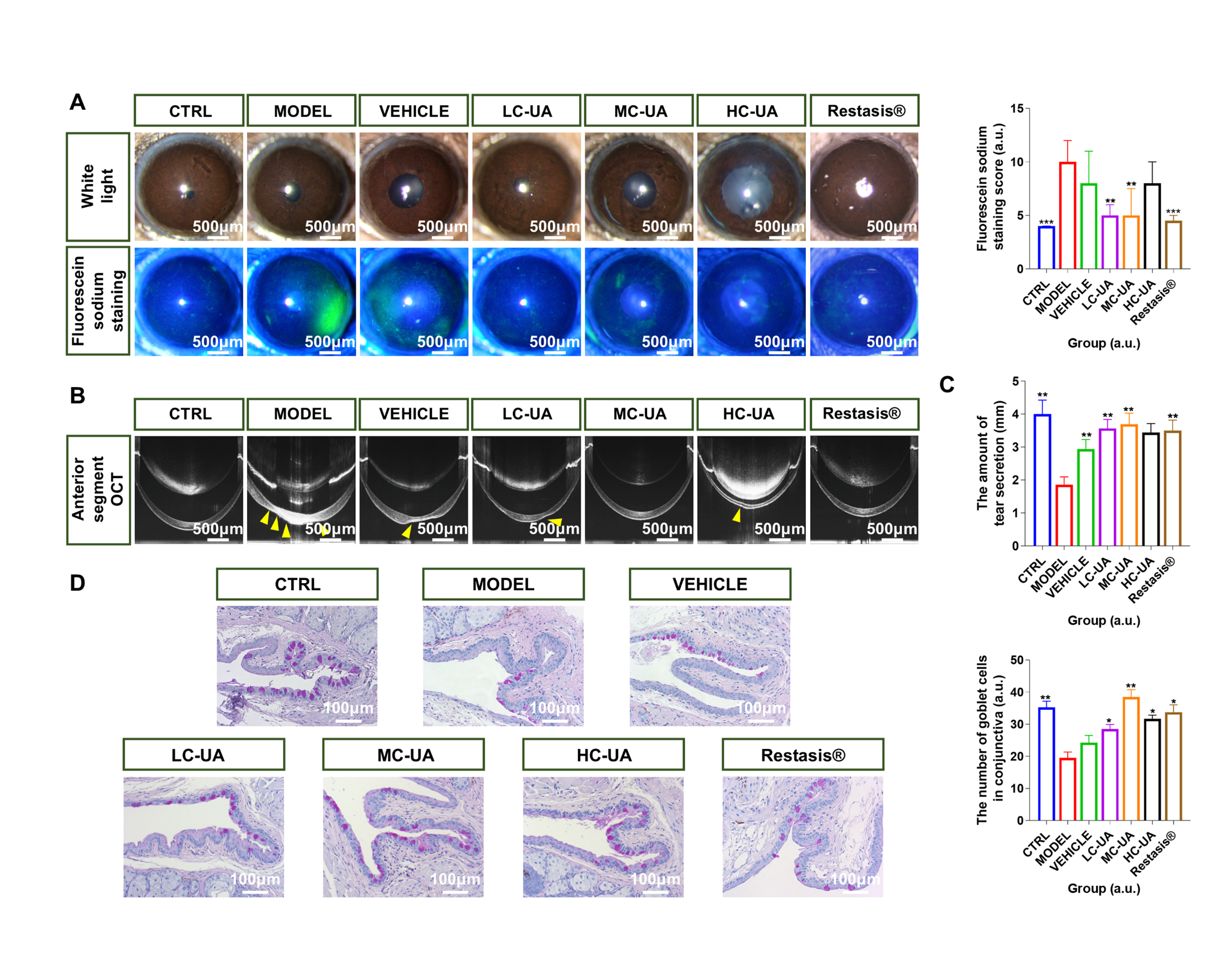


**Fig. S8. Ursolic acid (UA) relieves dry eye *in vivo*.** (**A**) Corneal fluorescence staining under slit lamp and evaluated scores quantification (*n* = 12 per group). (**B**) Anterior segment optical coherence tomography (OCT) images. (**C**) Tear secretion (*n* = 8 per group). (**D**) Periodic Acid-Schiff (PAS) staining of conjunctival goblet cells (GCs) and counting quantification (*n* = 8 per group). Data of (**A**) are expressed as medium ± interquartile range (IQR). The other data are expressed as mean ± Standard Error of the Mean (SEM).  ^*^*P* < 0.05, ^**^*P* < 0.01, and ^***^*P* < 0.001, comparison between the specified group and the model group. CTRL: control groups; MODEL: model groups; VEHICLE: vehicle groups; LC-UA: low concentration of UA; MC-UA: medium concentration of UA; HC-UA: high concentration of UA.


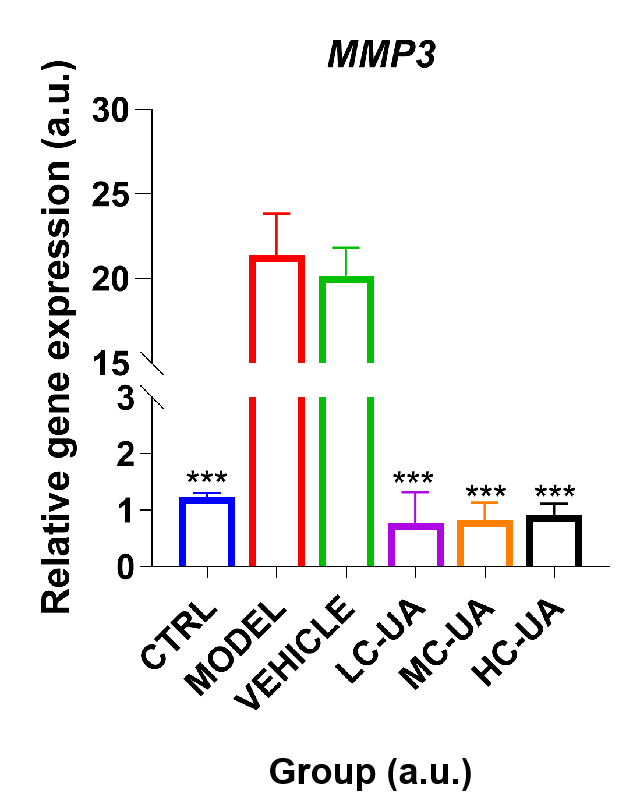


**Fig. S9.** **Ursolic acid (UA) protects cornea from desiccating stress.** Corneal matrix metalloproteinase-3 (*MMP3*) relative gene expression. Data are expressed as mean ± Standard Error of the Mean (SEM). ****P* < 0.001, comparison between the specified group and the model group. CTRL: control groups; MODEL: model groups; VEHICLE: vehicle groups; LC-UA: low concentration of UA; MC-UA: medium concentration of UA; HC-UA: high concentration of UA.

**
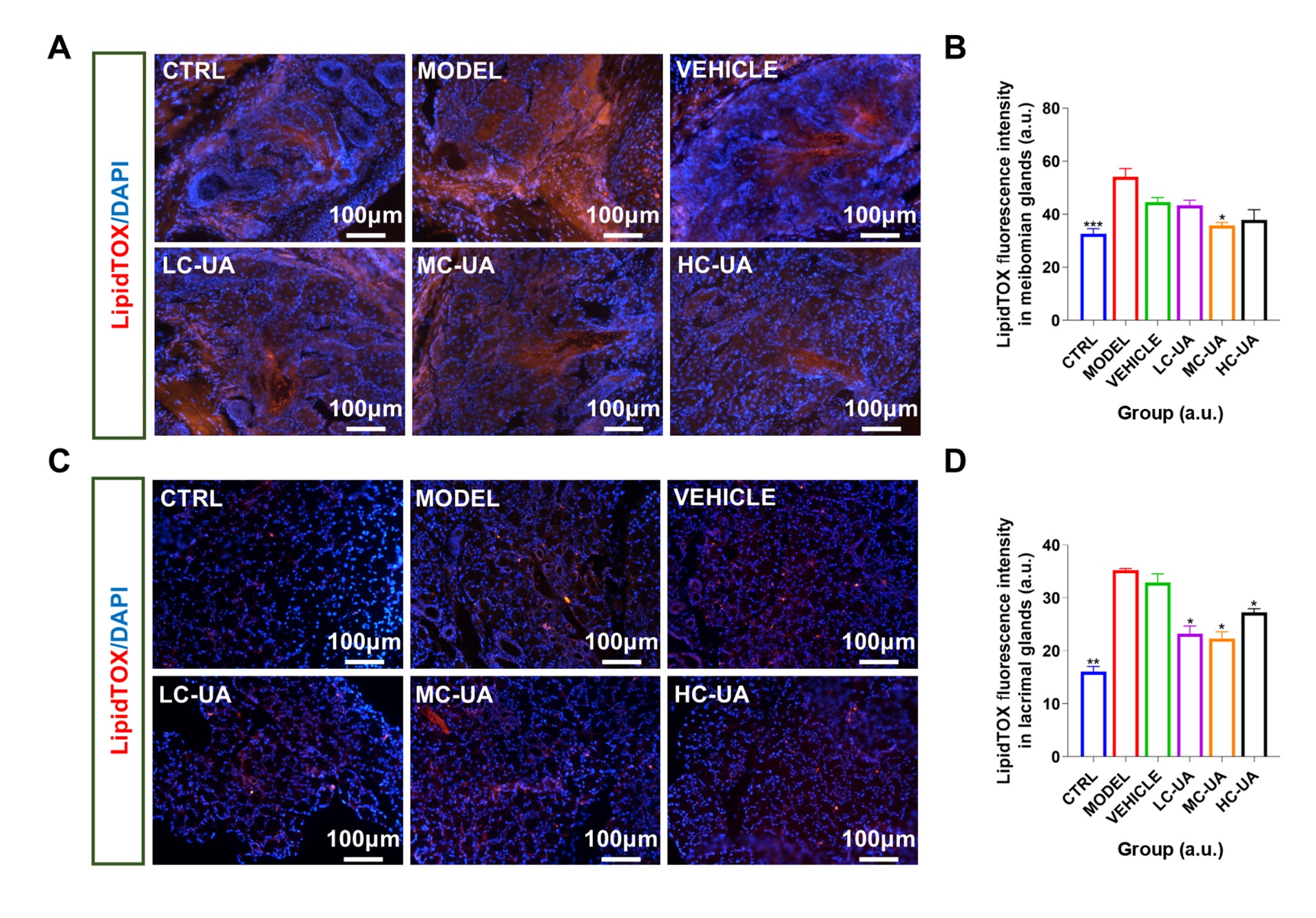
**

**Fig. S10. Ursolic acid (UA) alleviates abnormal lipid storage of meibomian glands and lacrimal glands.** (**A**) LipidTOX staining of the meibomian glands. (**B**) Quantification immunofluorescence intensity of LipidTOX staining in meibomian glands (*n* = 4 per group). (**C**) LipidTOX staining of the lacrimal glands. (**D**) Quantification immunofluorescence intensity of LipidTOX staining in Lacrimal glands (*n* = 3 per group). Data are expressed as mean ± Standard Error of the Mean (S.E.M.). **P* < 0.05, ***P* < 0.01 and ****P*< 0.001, comparison between the specified group and the model group. CTRL: control groups; MODEL: model groups; VEHICLE: vehicle groups; LC-UA: low concentration of Ursolic acid; MC-UA: medium concentration of Ursolic acid; HC-UA: high concentration of Ursolic acid.


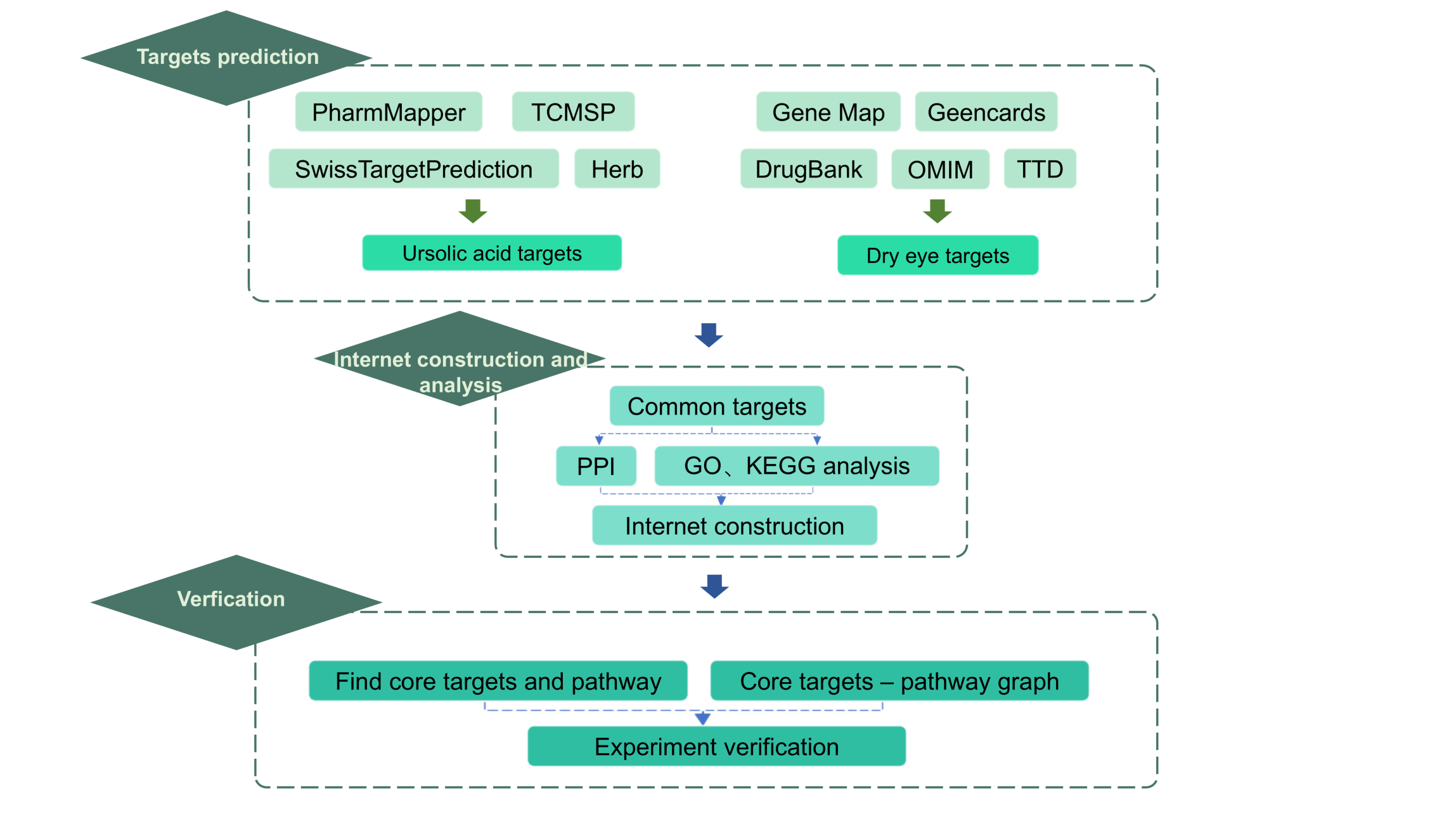


**Fig. S11.** **Ursolic acid (UA) and dry eye network construction of core targets between UA and dry eye (DE).** Analysis and experimental validation of the overall framework of network pharmacology. TCMSP: Traditional Chinese Medicine Systems Pharmacology Database and Analysis Platform; OMIM: Online Mendelian Inheritance in Man; TTD: Therapeutic Target Database; PPI: Protein-protein interaction; GO: Gene Ontology; KEGG: Kyoto Encyclopedia of Genes and Genomes.


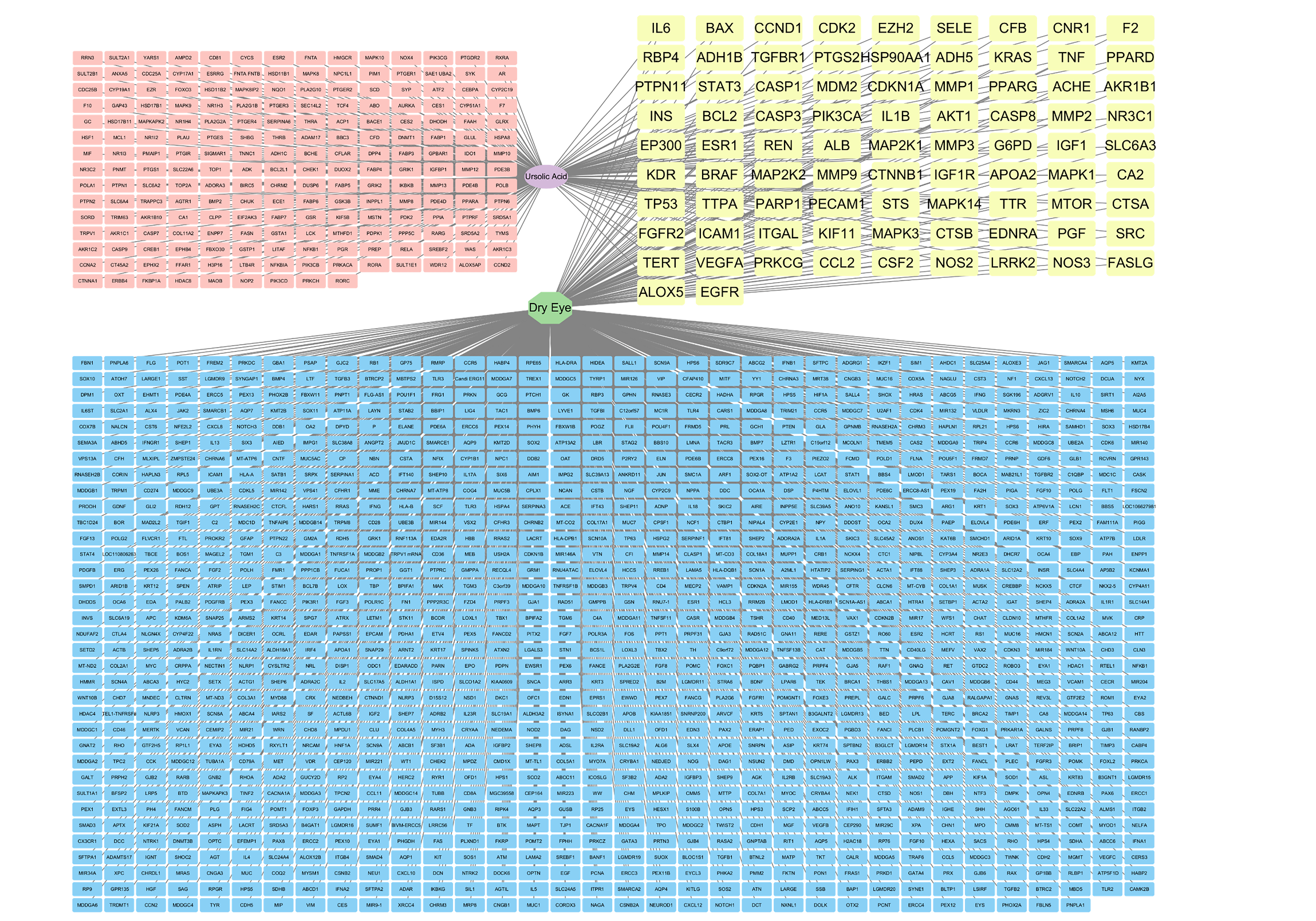


**Fig. S12.** **Visualization of intersecting targets.** The pink part is the non-intersecting **Ursolic acid (UA)** targets of UA and dry eye (DE), the blue part shows the non-intersecting DE targets of UA and DE, and the yellow part is the intersecting targets of UA and DE.


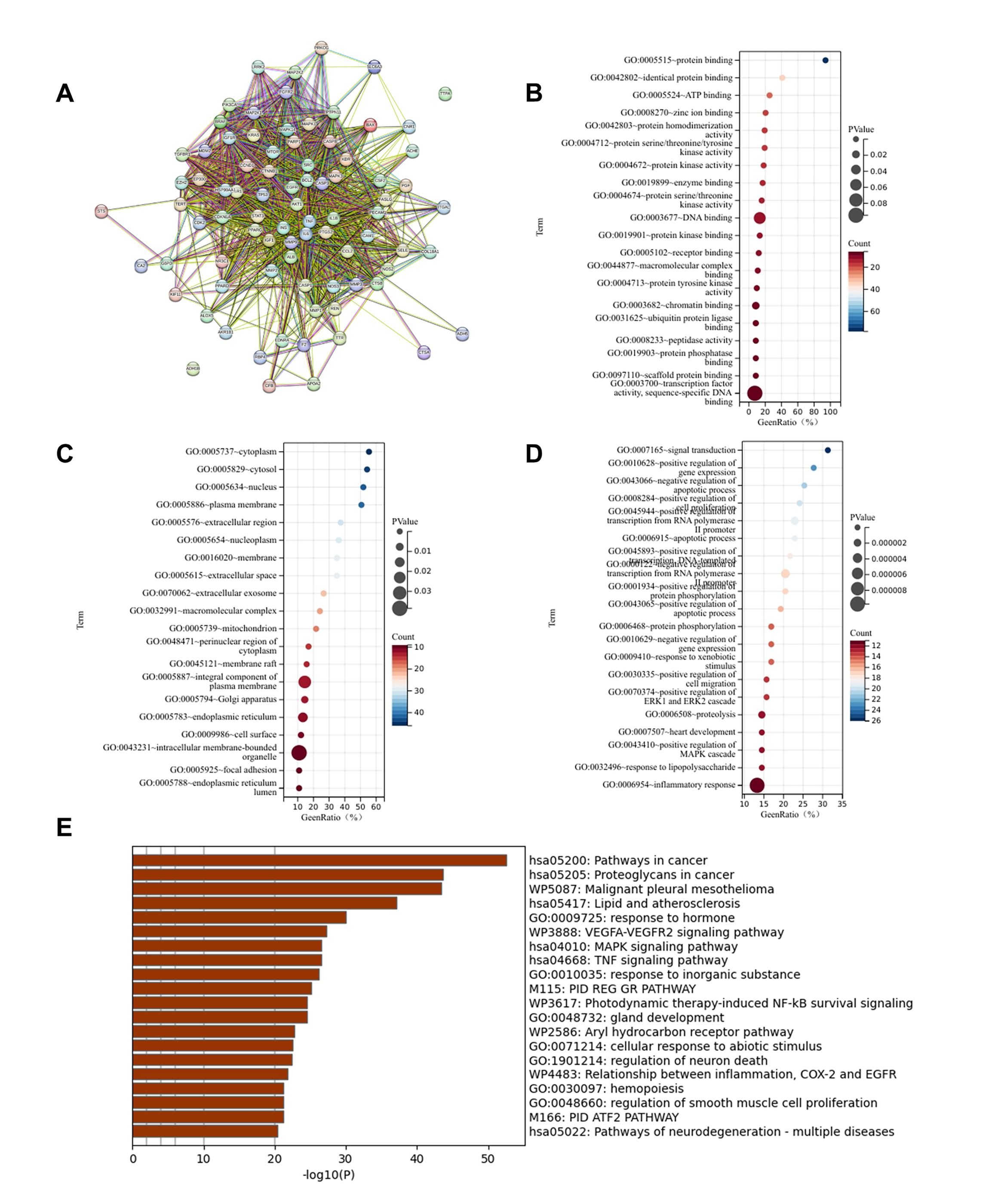


**Fig. S13.** **Ursolic acid (UA) and dry eye network construction of core targets between UA and dry eye (DE).** (**A**) Protein-protein interaction (PPI) network analysis to screen core targets. (**B -D**) GO analysis, from left to right, molecular function (MF), cellular component (CC) and biological process (BP). (**E**) Metascape platform core pathway enrichment analysis.


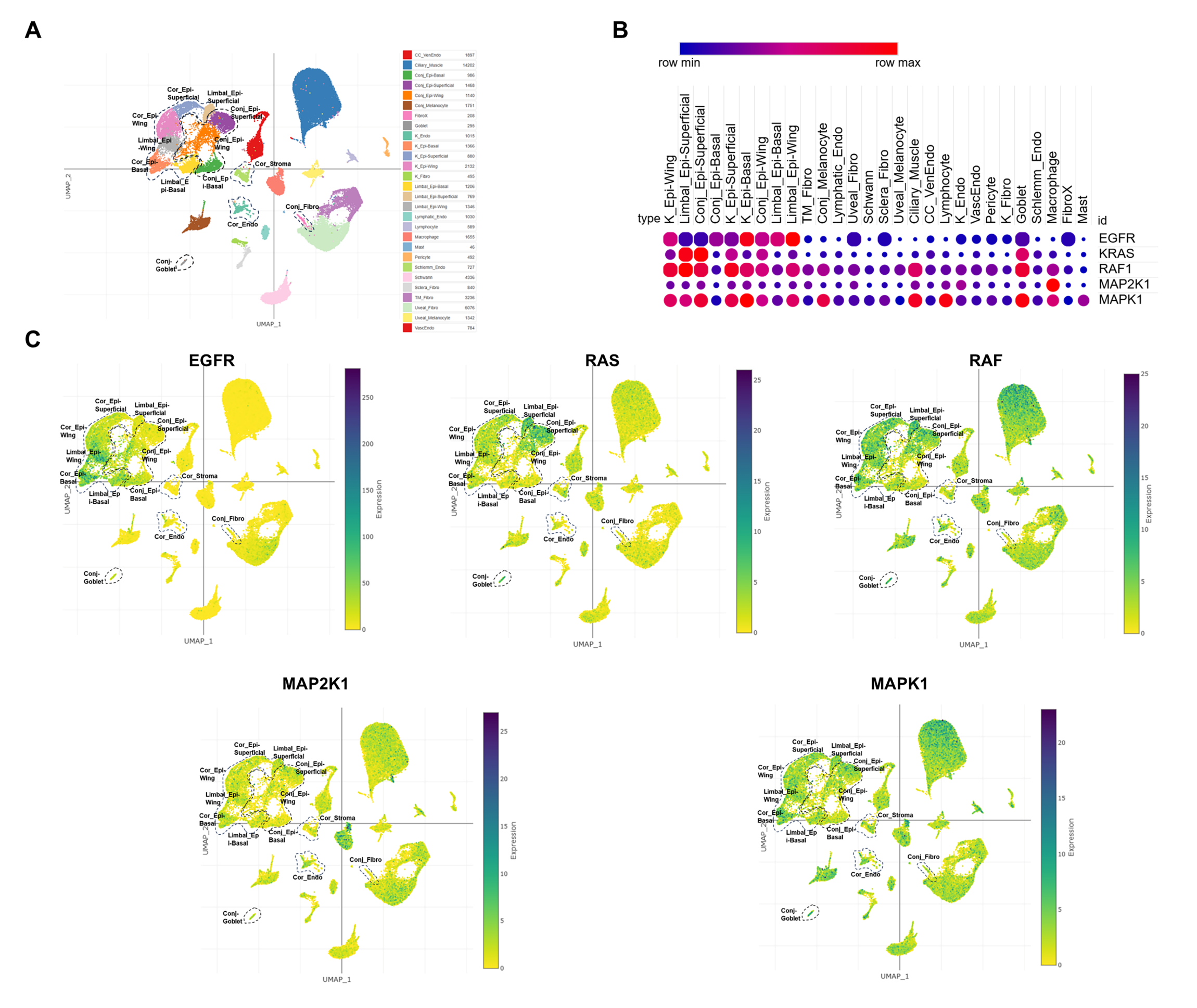


**Fig. S14. Single cell sequencing database analysis.** (**A**) Distribution of cell cluster in ocular anterior segment. (**B**) Comparison of Epidermal Growth Factor Receptor (EGFR), Rat Sarcoma (RAS), Fibrosarcoma (RAF), Mitogen-Activated Protein Kinase Kinase 1 (MAP2K1), and Mitogen-Activated Protein Kinase 1 (MAPK1) expression in ocular Anterior Segment. (**C**) Distribution and quantitative expression of EGFR, RAS, RAF, MAP2K1 and MAPK1 in ocular anterior segment. Cor: cornea; Epi: epithelium; Endo: endothelium; TA: transit amplify; Fibro: fibroblast.


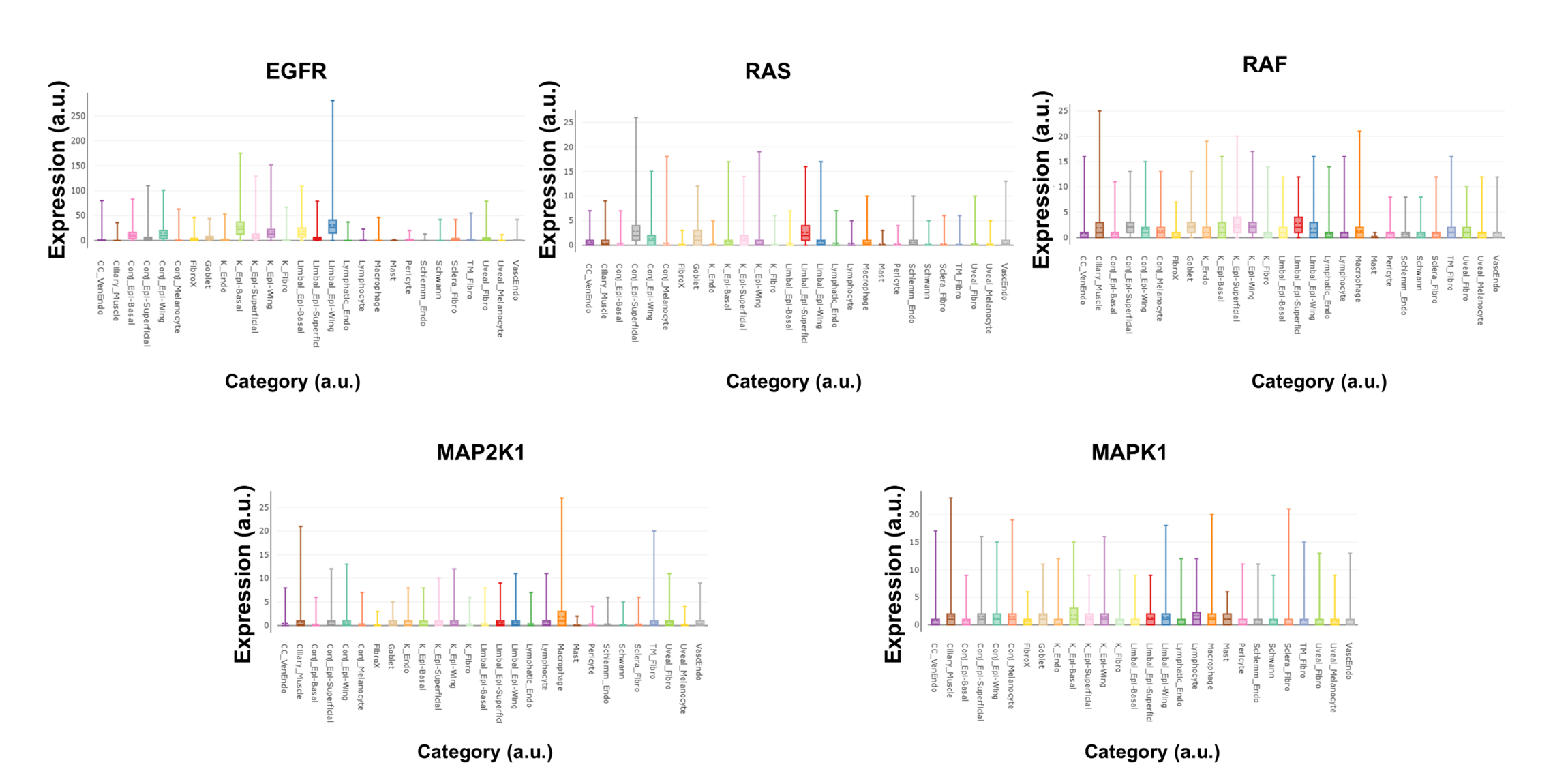


**Fig. S15. Single cell sequencing database analysis.** Quantitative expression of Epidermal Growth Factor Receptor (EGFR), Rat Sarcoma (RAS), Fibrosarcoma (RAF), Mitogen-Activated Protein Kinase Kinase 1 (MAP2K1), and Mitogen-Activated Protein Kinase 1 (MAPK1) in ocular anterior segment.


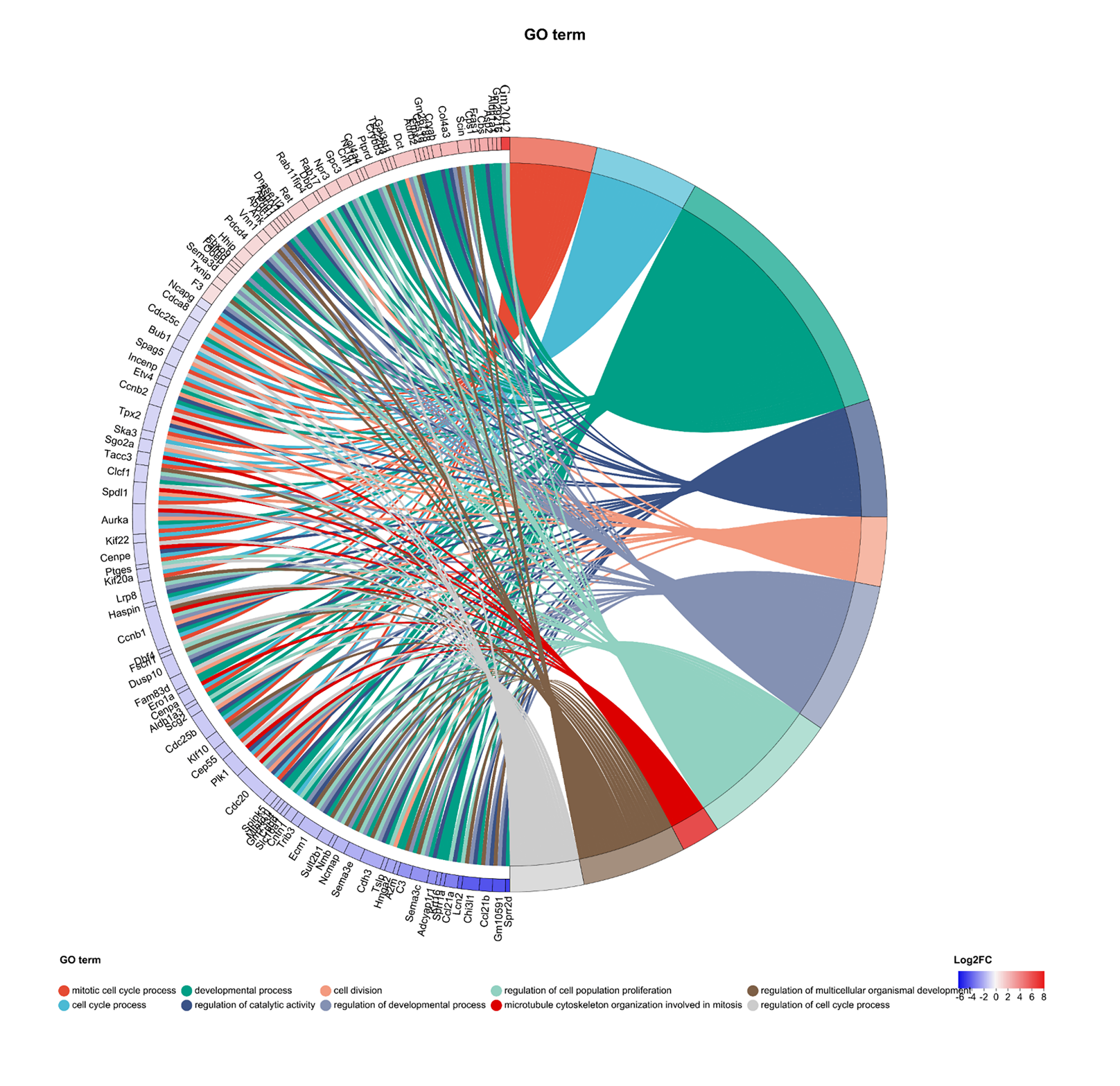


**Fig. S16. RNA sequencing analysis of the cornea treated with Ursolic acid (UA).** String diagram visualizing enriched gene functions and pathways from the Gene Ontology (GO) database.


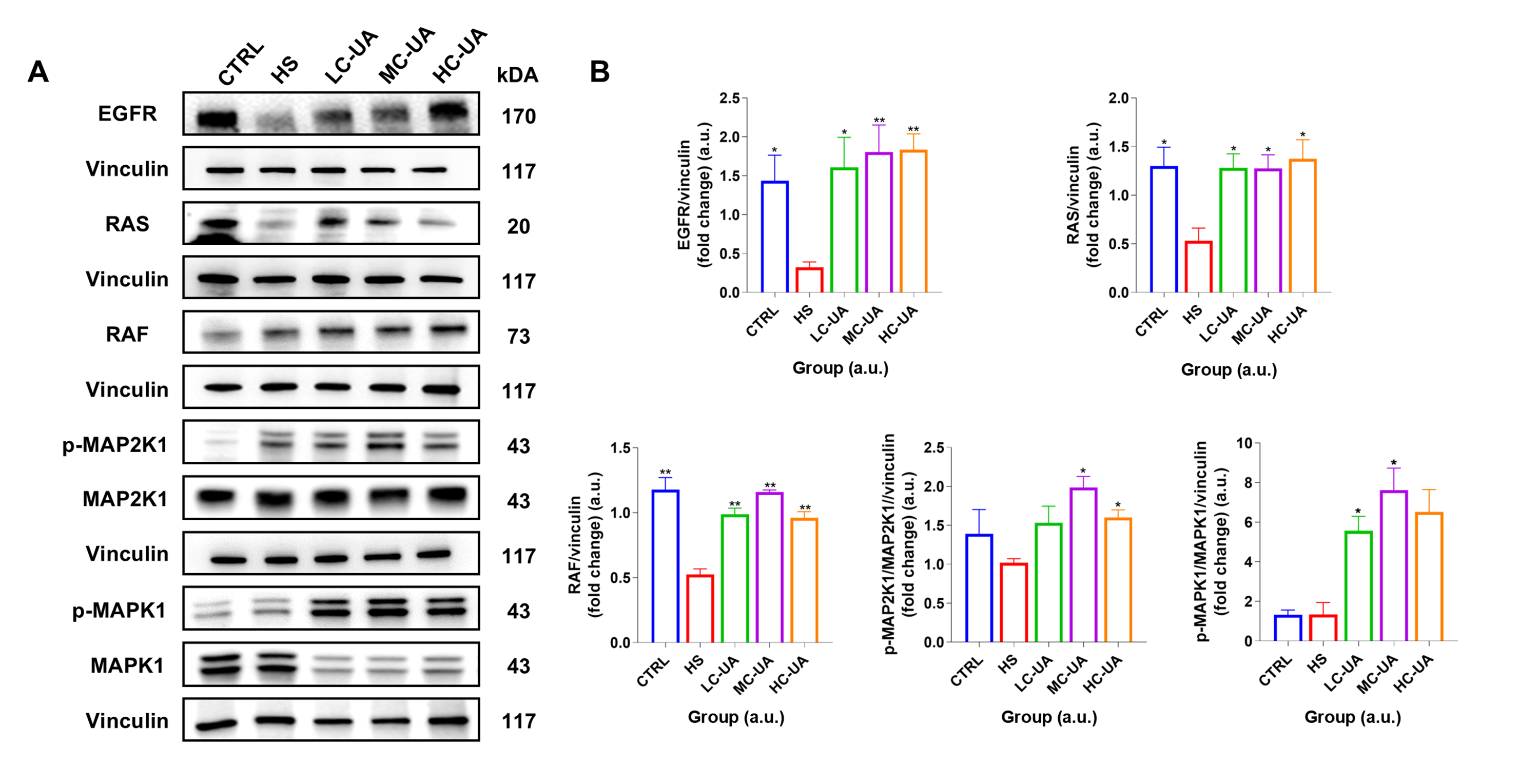


**Fig. S17**. **Ursolic acid (UA) alleviates dry eye via Epidermal Growth Factor Receptor (EGFR) / Rat Sarcoma (RAS) / Rapidly Accelerated Fibrosarcoma (RAF) / Mitogen-Activated Protein Kinase Kinase 1 (MAP2K1) / Mitogen-Activated Protein Kinase 1 (MAPK1) signaling pathway.** (**A**) Western Blot (WB) images of EGFR, RAS, RAF, p-MAP2K1, MAP2K1, MAPK1 and p-MAPK1 in HCEs. (**B**) Quantification of EGFR, RAS, RAF, p-MAP2K1/MAP2K1 and p-MAPK1/MAPK1 protein levels in HCEs (*n* = 5–9 per group). Data are expressed as mean ± Standard Error of the Mean (SEM). **P* < 0.05 and ***P*< 0.01, comparison between the specified group and the HS group. CTRL: control groups; MODEL: model groups; VEHICLE: vehicle groups; LC-UA: low concentration of UA; MC-UA: medium concentration of UA; HC-UA: high concentration of UA.


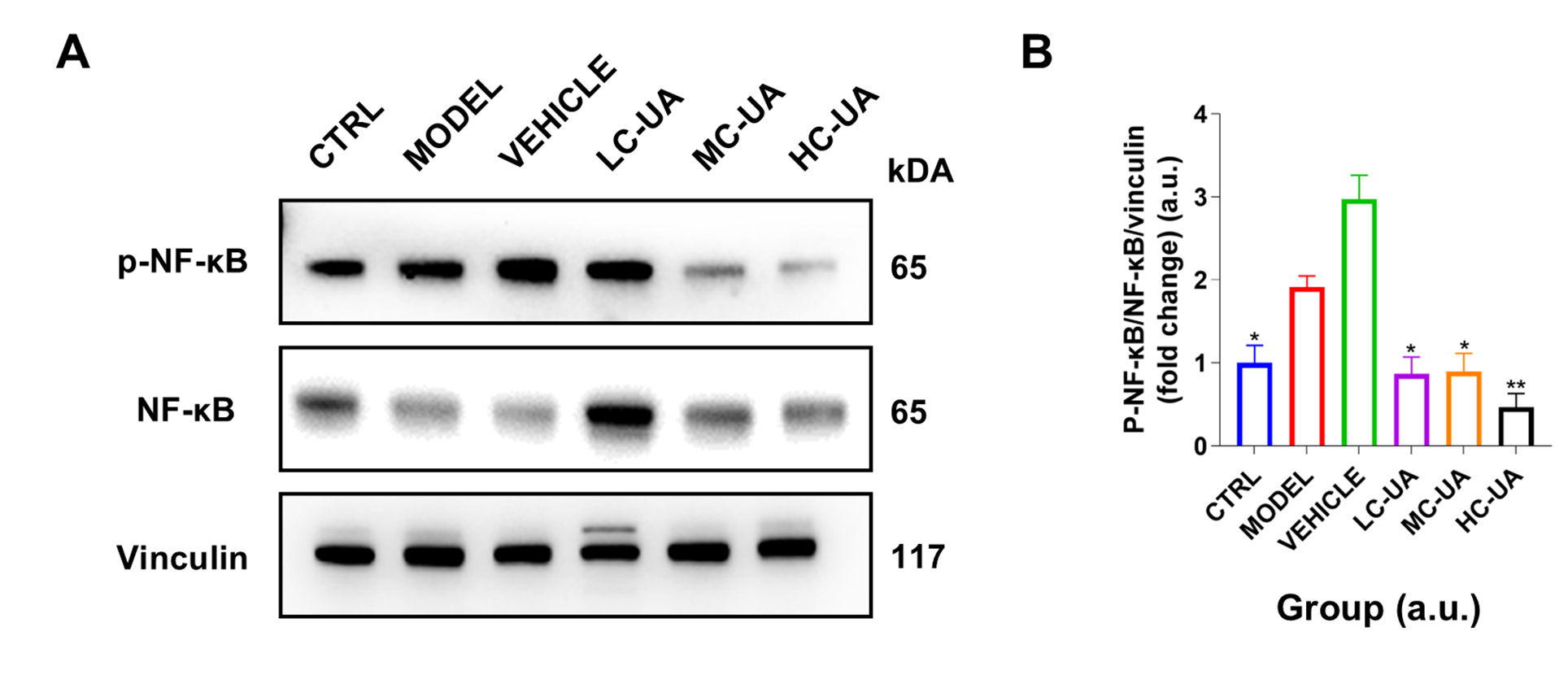


**Fig. S18. WB detection of Nuclear Factor Kappa-light-chain-enhancer of activated B (NF-κB) and p-NF-κB in the** **conjunctiva.** (**A**) Western Blot (WB) images of p-NF-κB, NF-κB and Vinculin in conjunctiva. (**B**) Quantification protein level of p-NF-kB/ NF-kB in conjunctiva (*n* = 6 per group). Data are expressed as mean ± Standard Error of the Mean (SEM). **P* < 0.05 and ***P* < 0.01, comparison between the specified group and the model group. CTRL: control groups; MODEL: model groups; VEHICLE: vehicle groups; LC-UA: low concentration of UA; MC-UA: medium concentration of UA; HC-UA: high concentration of UA.
