# Supplementary figures and images for: Ursolic acid ameliorates ocular surface dysfunction in dry eye via targeting EGFR/RAS/RAF/MAP2K1/MAPK1 pathway
Source: J Pharm Anal. 2025 Apr 3;15(11):101294. doi: 10.1016/j.jpha.2025.101294 (PMC12702019; doi:10.1016/j.jpha.2025.101294)

**Figure4 WB conjunctiva**


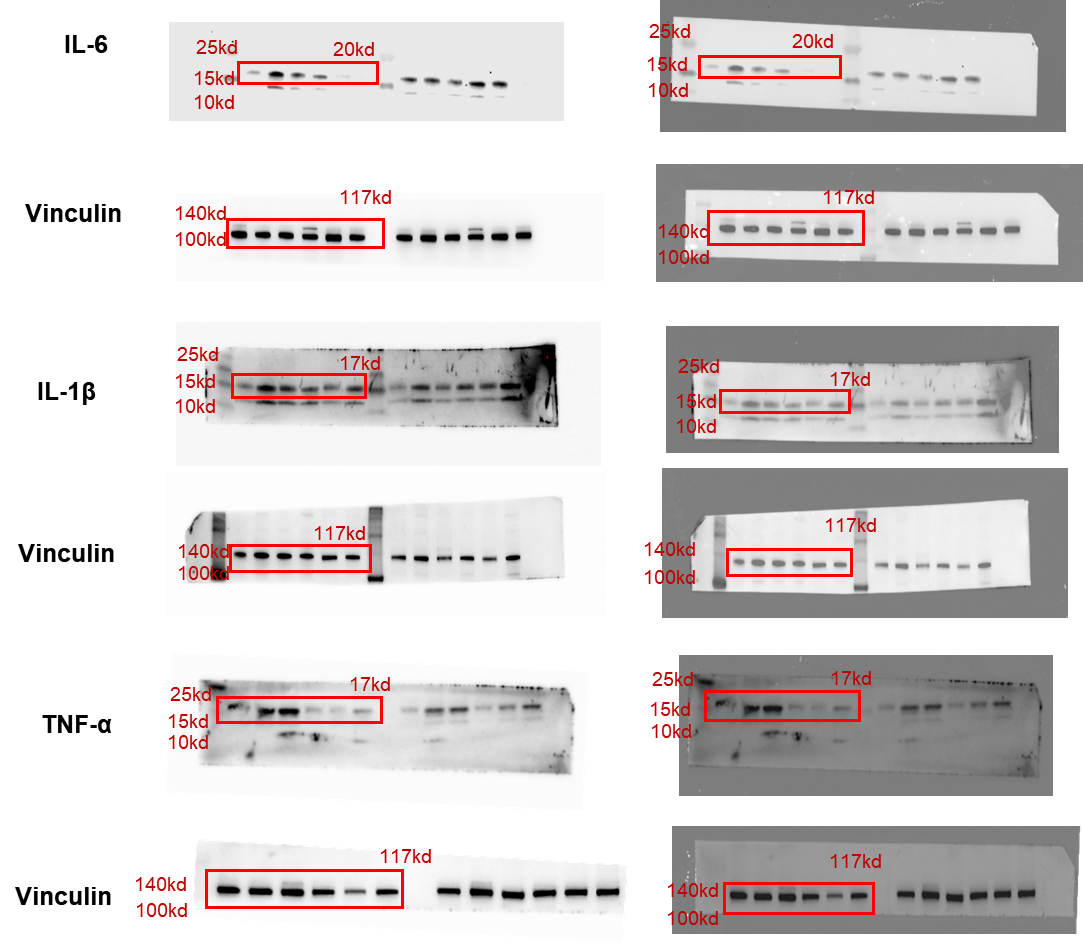


**Figure9 WB cornea**

**
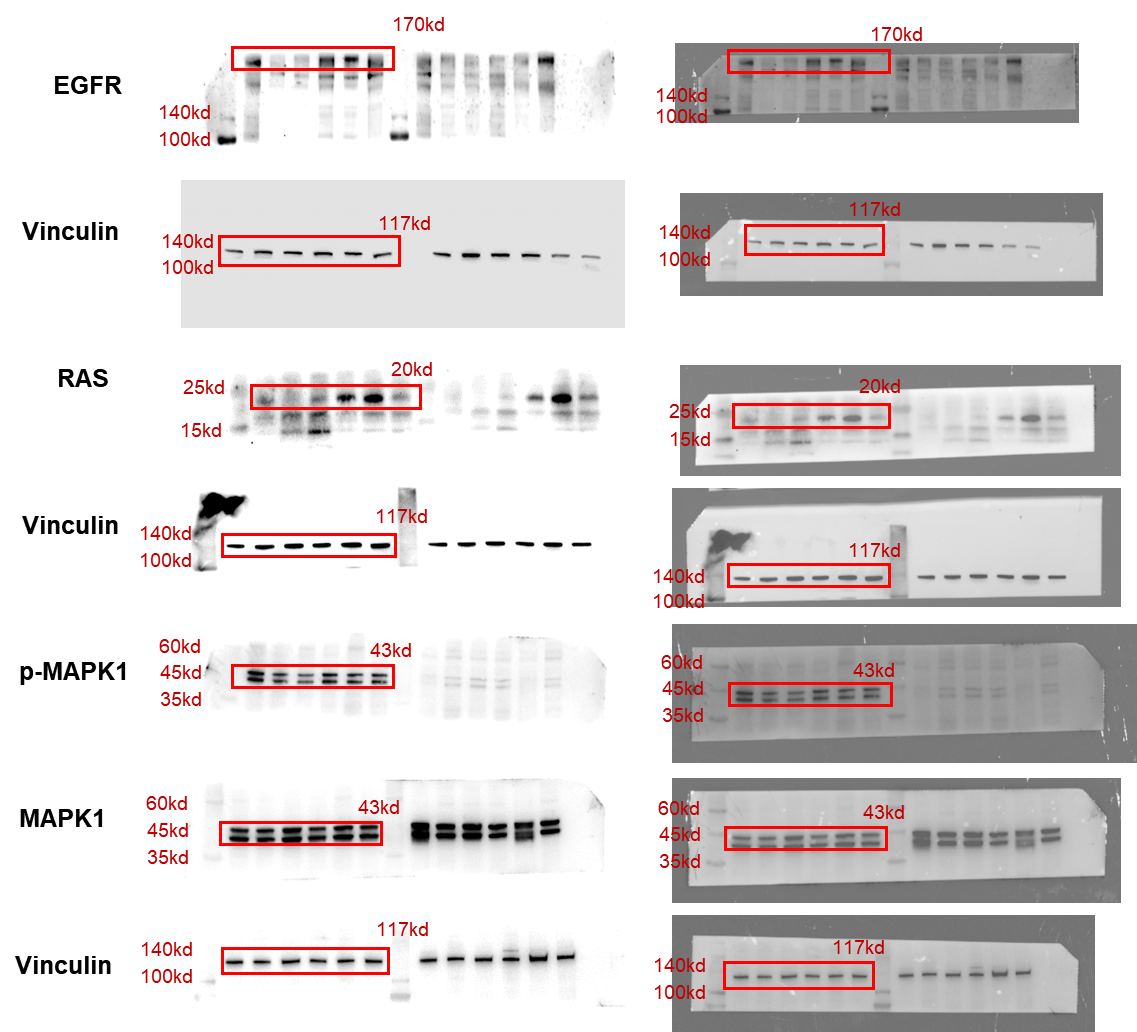
**

**
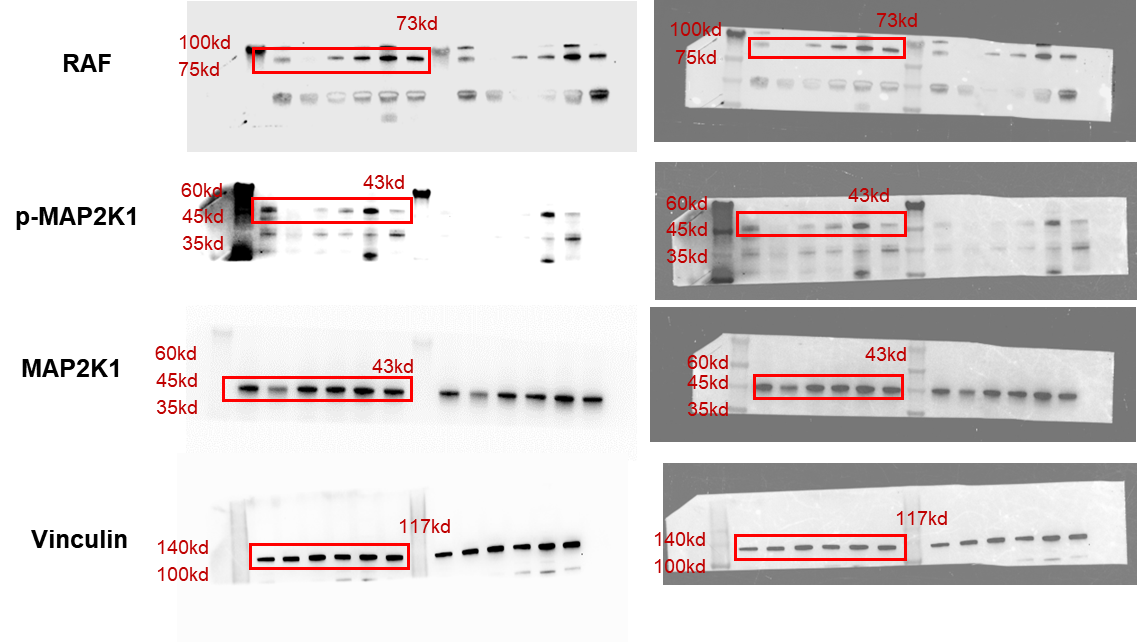
**

**Figure S17 HCE WB**

**
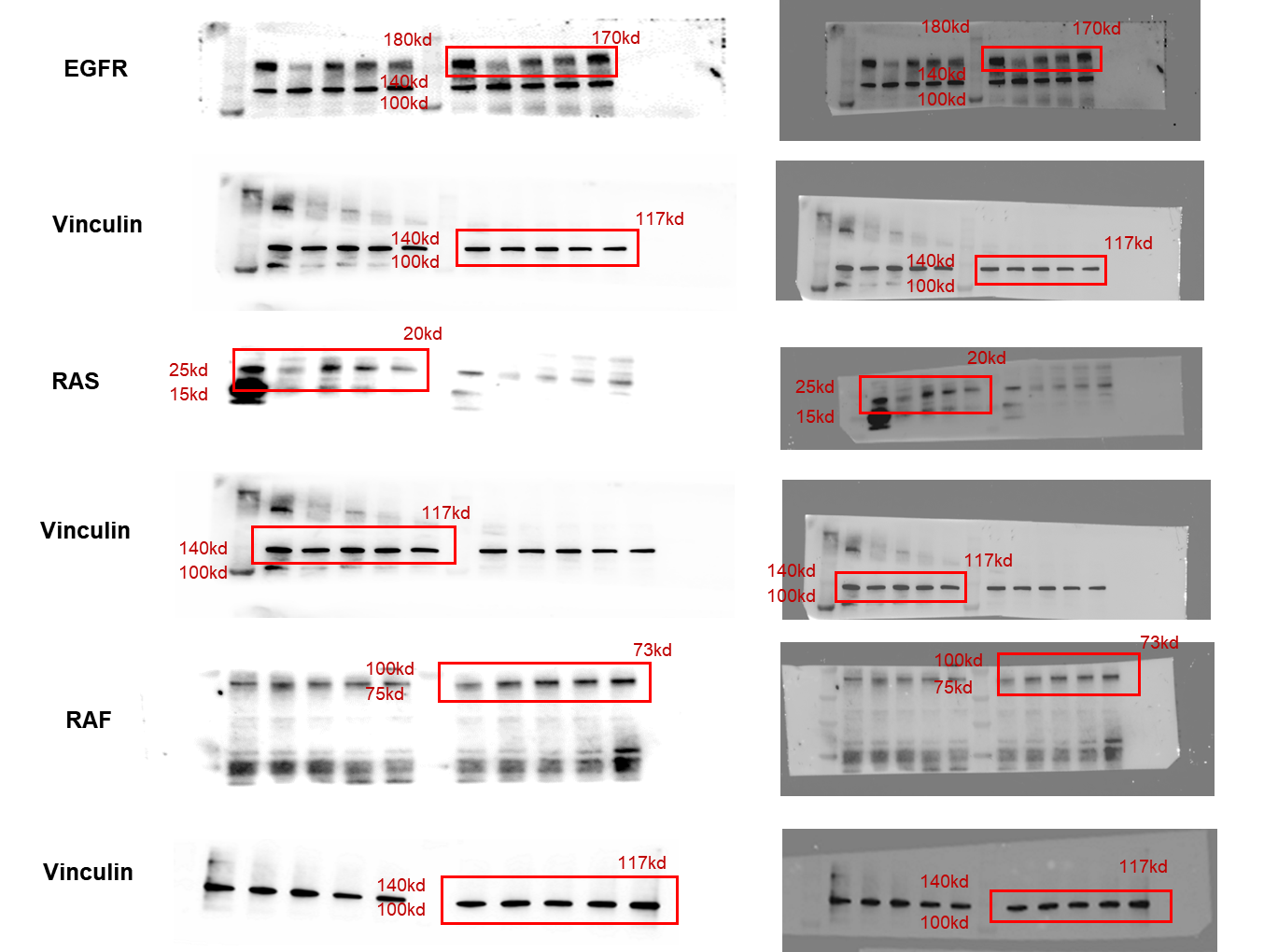
**

**
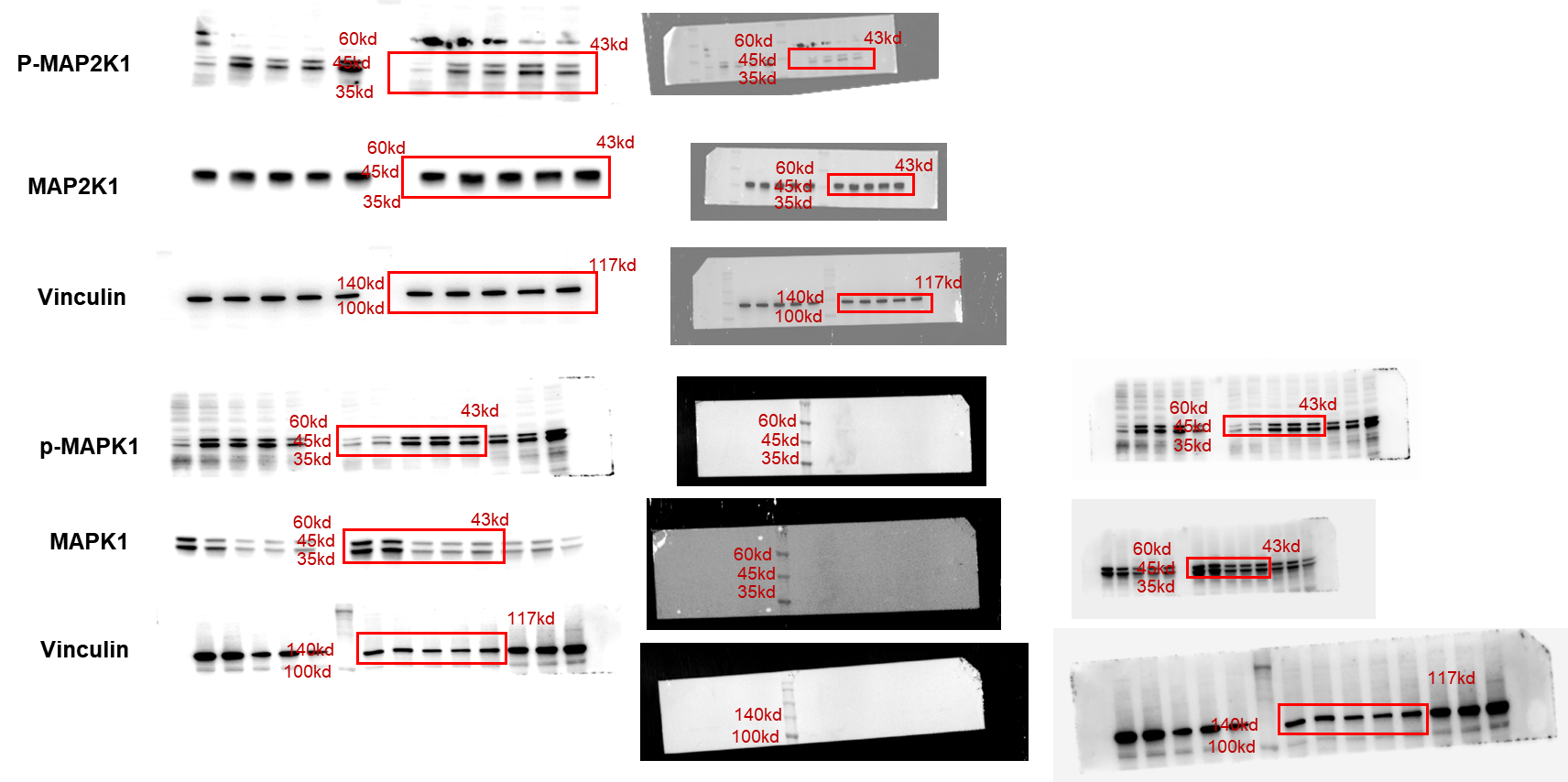
**

**Figure S18 conjunctiva**

**
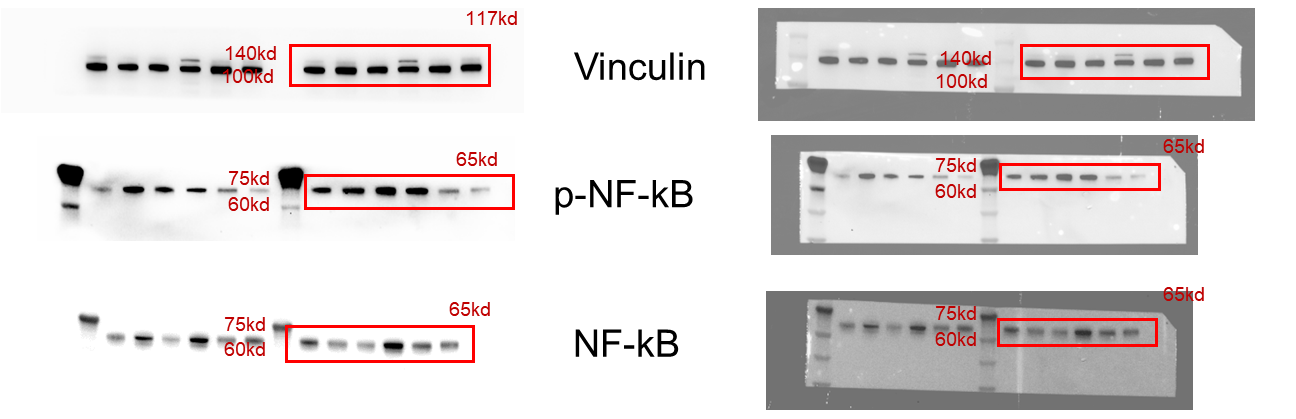
**

Supplement: Multimedia component 1 [file mmc1.zip › JPHA_101294 Supplementary Data/Original Data.docx]
